# Modulating *in vitro* lung fibroblast activation via senolysis of senescent human alveolar epithelial cells

**DOI:** 10.18632/aging.205994

**Published:** 2024-06-29

**Authors:** Joseph S. Spina, Tracy L. Carr, Lucy A. Phillips, Heather L. Knight, Nancy E. Crosbie, Sarah M. Lloyd, Manisha A. Jhala, Tony J. Lam, Jozsef Karman, Meghan E. Clements, Tovah A. Day, Justin D. Crane, William J. Housley

**Affiliations:** 1 AbbVie Bioresearch Center, Worcester, MA 01605, USA; 2 Department of Biology, Northeastern University, Boston, MA 02115, USA; 3 AbbVie Inc., North Chicago, IL 60064, USA; 4 Current address: Merck, Cambridge, MA 02141, USA; 5 Current address: Pfizer Inc., Cambridge, MA 02139, USA

**Keywords:** cellular senescence, fibrosis, senolytic, senomorphic, SASP, alveolar epithelial cell

## Abstract

Idiopathic pulmonary fibrosis (IPF) is an age-related disease with poor prognosis and limited therapeutic options. Activation of lung fibroblasts and differentiation to myofibroblasts are the principal effectors of disease pathology, but damage and senescence of alveolar epithelial cells, specifically type II (ATII) cells, has recently been identified as a potential trigger event for the progressive disease cycle. Targeting ATII senescence and the senescence-associated secretory phenotype (SASP) is an attractive therapeutic strategy; however, translatable primary human cell models that enable mechanistic studies and drug development are lacking. Here, we describe a novel system of conditioned medium (CM) transfer from bleomycin-induced senescent primary alveolar epithelial cells (AEC) onto normal human lung fibroblasts (NHLF) that demonstrates an enhanced fibrotic transcriptional and secretory phenotype compared to non-senescent AEC CM treatment or direct bleomycin damage of the NHLFs. In this system, the bleomycin-treated AECs exhibit classical hallmarks of cellular senescence, including SASP and a gene expression profile that resembles aberrant epithelial cells of the IPF lung. Fibroblast activation by CM transfer is attenuated by pre-treatment of senescent AECs with the senolytic Navitoclax and AD80, but not with the standard of care agent Nintedanib or senomorphic JAK-targeting drugs (e.g., ABT-317, ruxolitinib). This model provides a relevant human system for profiling novel senescence-targeting therapeutics for IPF drug development.

## INTRODUCTION

Idiopathic pulmonary fibrosis (IPF) is an age-related, chronic, progressive disease of the lung with unknown etiology and limited treatment options. To date, only two small molecule drugs, Nintedanib and Pirfenidone, have been approved for IPF, while Tocilizumab, an anti-IL-6 biologic, is approved for systemic sclerosis-associated interstitial lung disease (SSc-ILD) [1, 2], a related pathology. While these drugs may slow the progression of disease, patients only survive a median of 3–5 years post-diagnosis [1], highlighting an unmet need for therapeutic options that target the underlying pathology of IPF.

Chronically activated fibroblasts and differentiation of myofibroblasts in the lung following tissue damage are regarded as primary pathogenic features of IPF [3, 4]. These activated fibroblasts secrete excessive matrix that becomes overly stiff and impairs proper gas exchange in the alveoli, leading to chronic loss of lung function and diminished physical abilities of the patient [3]. While the current clinical therapies are thought to target broad anti-fibrotic mechanisms primarily within fibroblasts, such as TGF-β signaling [1, 3], recent single-cell RNA sequencing studies point to an aberrant population of epithelial cells as potential initiators of disease [5–7]. These cells originate as stem-cell like alveolar epithelial type II (ATII) cells, which, upon damage or repetitive microinjury, progress through an intermediate KRT8^+^ state, to become KRT5^+^ aberrant basal cells [5–9]. These damage-associated transient progenitor (DATP) intermediate states, termed pre-alveolar type-1 transitional cell state (PATS) or alveolar differentiation intermediates (ADI), represent senescence-associated differentiation disorder (SADD) and exhibit many hallmarks of cellular senescence and persist in the IPF lung [10].

Cellular senescence, the arrest of cell cycle progression of damaged or replication-exhausted cells [11], has been well documented in IPF patient lungs [12–17]. It is hypothesized that exceeding a pathogenic threshold of senescent cells can induce age-related diseases, such as IPF [18, 19]. These cells are highly secretory, releasing a potent milieu of pro-inflammatory cytokines, chemokines, growth factors, and matrix remodeling proteins, termed the senescence-associated secretory phenotype (SASP) [20]. Indeed, senescence, rather than depletion, of ATII cells was sufficient to drive progressive lung fibrosis in a Sin3a conditional loss-of-function mouse model [21]. Thus, with accumulated environmental damage and weakened immunosurveillance [22], older adults may be predisposed to surpass the pathogenic threshold of senescent cells in the lung and trigger progressive fibrotic disease.

Recent findings suggest that clearing senescent cells in bleomycin- and irradiation-induced murine lung fibrosis models with a combination of Dasatinib and Quercetin (DQ) [15] (see also [23]) or the BCL-2/xL inhibitor Navitoclax (ABT-263) [16, 24, 25] partially reverses disease pathology. Many existing *in vitro* methods to understand senescence mechanisms in the context of fibrosis pathogenesis rely on immortalized cell lines, primary cells from rodents, or are not amenable to profiling novel therapeutic mechanisms [12, 13, 26–28]. To enable drug development, complex *in vitro* systems that facilitate compound screening for senescent cell clearing and SASP inhibition in relevant cell types are needed.

In the current study, we confirm the presence of senescent cell populations within the human IPF lung, as well as assess primary cell reagents for sensitivity to senescent cell targeting therapies. To overcome the logistic challenge of sourcing diseased, primary human alveolar epithelial cells (AEC) from patients, we have developed a novel model using bleomycin to induce senescence in healthy AECs based on a previous method of senescence induction in the epithelial adenocarcinoma-like A549 cell line [27]. We reproduce, in an *in vitro* plate-based system of alveolar epithelial cell damage, a population of epithelial cell-derived aberrant cells, representative of those in the IPF lung, that express hallmarks of senescence [5–10]. Conditioned medium (CM) from these AECs induces significantly more pro-fibrotic protein secretion in NHLFs than non-senescent AEC control CM, and this damage is more pro-fibrotic than direct bleomycin injury of the NHLFs themselves.

This system is amenable to evaluating a variety of therapeutic mechanisms [29]: anti-fibrotic drugs such as standard of care Nintedanib, anti-inflammatory or senomorphic drugs that inhibit the SASP such as JAK1/2 inhibitor ruxolitinib [30], and importantly, senolytic agents such as Navitoclax. To investigate novel mechanisms of existing late-stage clinical assets, AD80, a multi-kinase inhibitor targeting RET (c-RET), BRAF, S6K, and SRC that is under investigation for the treatment of certain cancers, was included as a potential senolytic. AD80 has shown apoptosis-inducing effects in various cell types, particularly on cells in G_2_/M phase [31, 32], and was hypothesized to also impact injured AECs. ABT-317, a JAK1-selective inhibitor, was also tested as a potential senomorphic that is more selective than ruxolitinib [33]. We show that senolytic, but not senomorphic or anti-fibrotic, treatments of senescent alveolar epithelial cells prior to conditioned medium transfer attenuates the impact of the SASP on inducing collagen secretion from lung fibroblasts, contributing key proof-of-concept mechanistic data in a translatable, IPF-relevant human cellular system.

## RESULTS

### p16^ink4a^ is observed in the aberrant epithelium but not fibroblasts of human IPF lung tissues; similarly, senescence markers are not an inherent hallmark of primary IPF fibroblast cells *in vitro*

**Figure 1:**
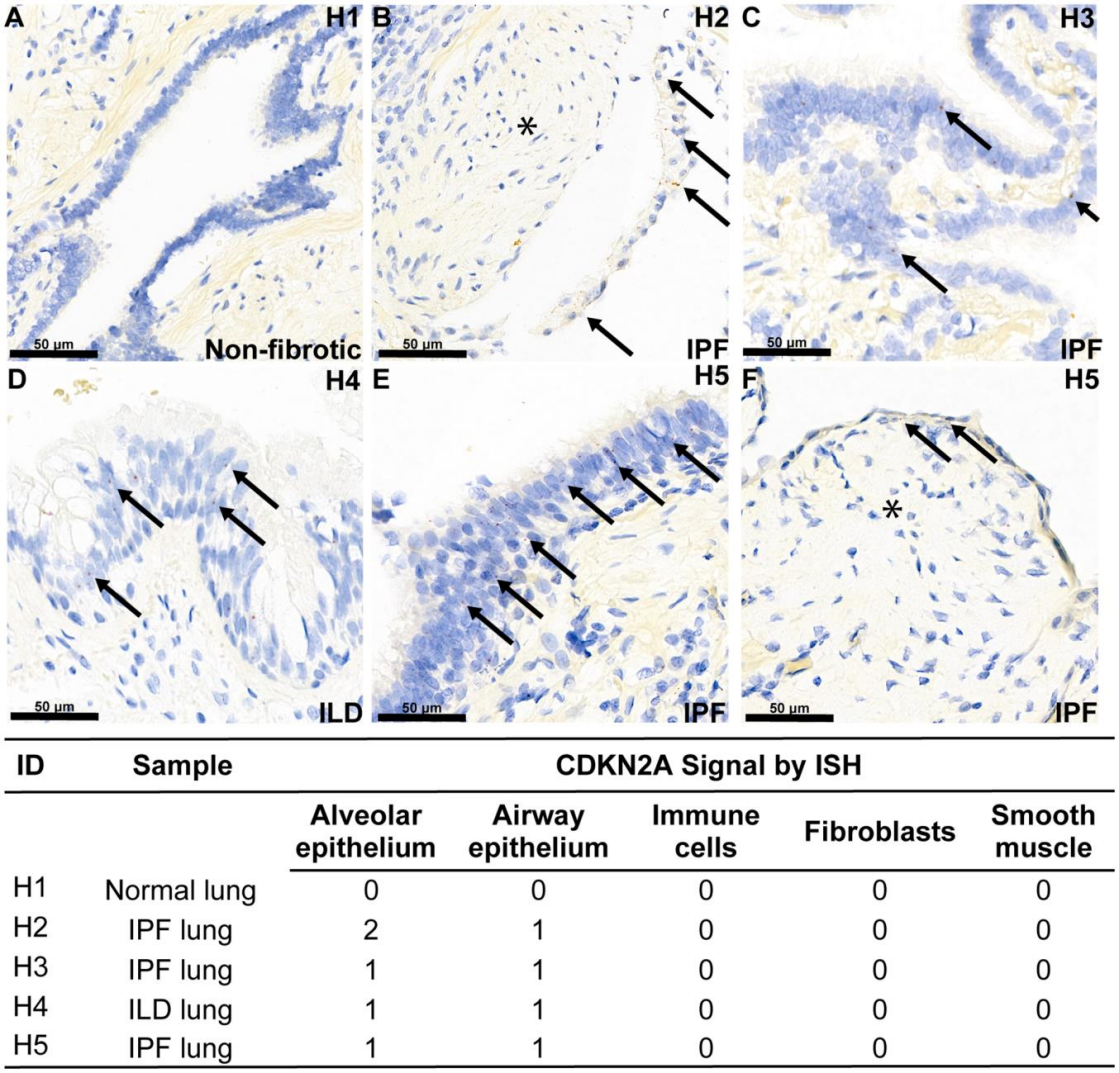
**Aberrant epithelial cell senescence is a hallmark of fibrotic lung disease.** RNA ISH was performed with a probe against transcript variant 1 of the human *Cdkn2a* gene locus, specifically detecting the p16^ink4a^-coding transcript, on histological slides from four human donors with end-stage interstitial lung disease or idiopathic pulmonary fibrosis and one human donor without lung disease. Demographic information is noted in Supplementary Table 1. Example red ISH puncta representing p16^ink4a^ transcripts are identified with an arrow, and areas of p16^ink4a^-negative fibroblastic foci are marked with an asterisk. Expression of p16^ink4a^ mRNA expression across cell types per donor using a semi-quantitative 0–3 scale is shown in the associated table. (**A**) No *Cdkn2a* mRNA is observed in bronchiolar epithelium of normal lung, (**B**–**F**) *Cdkn2a* mRNA is present in the aberrant epithelium overlying fibroblastic foci (asterisk) and in the aberrant epithelium lining honeycomb cysts in diseased lung.

To confirm that cellular senescence is found in fibrotic interstitial lung disease in humans, we performed RNA *in situ* hybridization (ISH) with a probe specifically designed for the p16^ink4a^ transcript variant of the *Cdkn2a* gene locus in diseased lung. There are several transcriptional splice variants of the *Cdkn2a* locus and commercial antibody reagents exhibit poor isoform specificity, therefore we chose RNA detection to more accurately measure the p16^ink4a^ isoform (transcript variant 1) of *Cdkn2a* that is specifically associated with permanent cell cycle arrest [34]. All interstitial lung disease patient tissue sections showed mild expression of *Cdkn2a* in the aberrant and airway epithelial cells lining the honeycomb cysts, but notably, not in fibroblasts in the fibroblastic foci, which define the site of active matrix deposition (Figure 1). A quality control probe against *Ppib* transcript was included, which confirms that the absence of signal in the fibroblasts is not due to diminished RNA quality (Supplementary Figure 1). The age-matched, non-fibrotic lung section showed no *Cdkn2a* mRNA, and as expected, did not contain aberrant epithelial cells owing to the absence of disease (Figure 1). Demographic information for lung tissue donors can be found in Supplementary Figure 1.

**Figure 2:**
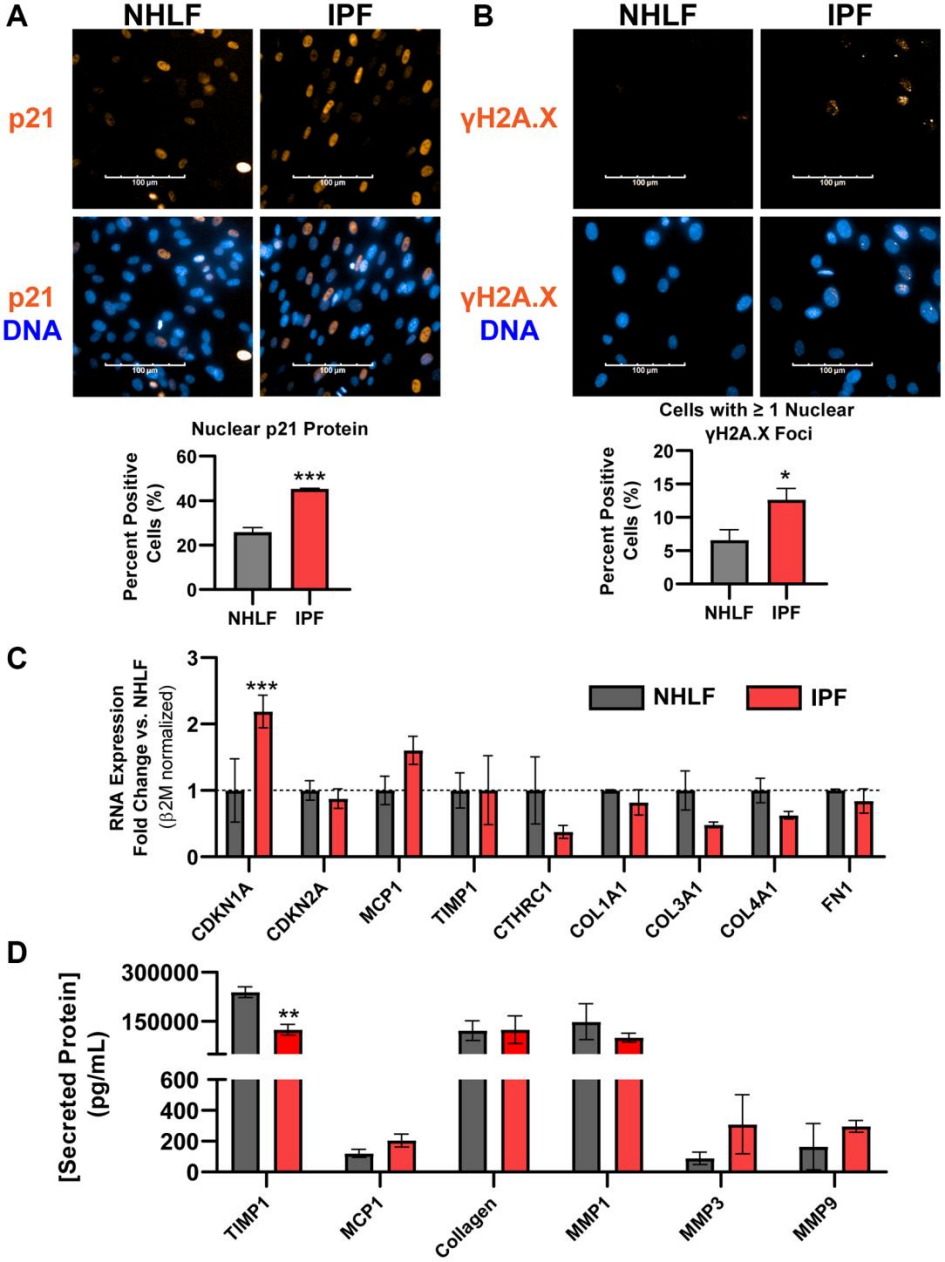
**DNA damage is a hallmark of commercial primary IPF fibroblasts *in vitro*, but not *Cdkn2a*/p16^ink4a^ expression or a fibrotic secretome**. (**A**) Primary NHLF and IPF cells (*n* = 3 different donors, each with 2 technical replicates) were cultured at the same density overnight on standard tissue culture plates in low-serum growth medium and were subsequently fixed. Immunocytochemistry was performed to fluorescently label nuclei (blue) and p21^Waf1/Cip1^ (orange). Images were acquired with a 40X water objective using an Operetta High Content Screening instrument and intensity of nuclear p21^Waf1/Cip1^ was quantified and calculated as a positive percentage of each population. (**B**) NHLF and IPF cells were treated and imaged as in (**A**), and immunocytochemistry was performed to fluorescently label nuclei (blue) and DNA damage via nuclear γH2A.X (Ser139) foci (orange). The number of nuclear foci per cell was quantified and cells with one or more were reported as positive. (**C**) Following overnight culture in standard tissue culture plates, NHLF and IPF cells were lysed, and qPCR was performed. Expression of senescence and fibrosis-related matrix and secreted factor genes was assessed, and data were normalized to *β2m* housekeeper expression using the 2^−ΔΔCt^ method versus NHLF cells. (**D**) Following overnight culture as in (**C**), cell culture supernatants were collected and assayed for determination of the concentration of common fibrosis-related secreted proteins by MSD kits. Statistical analysis was performed using an unpaired *t*-test (**A**, **B**) or a two-way ANOVA with a Bonferroni post-test (**C**, **D**) in GraphPad Prism: ^*^p < 0.05, ^**^p < 0.01, ^***^p < 0.001. Error bars represent standard error of the mean (SEM).

While expression of the permanent cell cycle inhibitor p16^ink4a^ transcript was absent in fibroblasts in human IPF histology, fibroblasts within the active fibroblastic foci are considered the primary drivers of excessive matrix deposition and overall disease progression [3]. Other markers of senescence, notably expression of the DNA damage marker γH2A.X and the associated cell cycle inhibitor p21^Waf1/Cip1^, were both significantly increased at the protein level in fixed IPF lung fibroblasts compared to normal human lung fibroblast (NHLF) cells by fluorescent high content imaging analysis (Figure 2A, 2B). Despite observing increased expression of *Cdkn1a*/p21^Waf1/Cip1^ and persistent DNA damage in IPF fibroblasts, *Cdkn2a*/p16^ink4a^ gene expression was not elevated (Figure 2C). Commercially available primary IPF fibroblast cells also did not show a reliable pro-fibrotic phenotype in culture. Specifically, transcripts of matrix proteins *Col1a1*, *Col3a1*, and *Col4a1*, *Fn1* as well as other fibrosis-related markers *Cthrc1* (ECM component), *Mcp1* (chemokine), and *Timp1* (inhibitor of MMP activity) were not significantly increased over NHLFs, despite the significantly elevated expression of *Cdkn1a* transcript (Figure 2C). Similarly, secreted fibrotic factors collagen, TIMP1, MMP1, MMP3, MMP9, and MCP1 showed no significant increases in IPF cells at the protein level (Figure 2D). The expression of *Cdkn1a* and *Cdkn2a* in primary IPF and NHLF cells from 11 and 5 additional donors, respectively, representing a variety of vendor sources, was assessed without the stress of culture conditions by lysing cells immediately after thawing from the cryovials. Significant variability in expression in both *Cdkn1a* and *Cdkn2a* was observed (Supplementary Figure 2), implying that donor-to-donor variability or differences in isolation and culture methods between vendors result in an unreliable senescent phenotype with commercial products. Taken together, IPF lung fibroblasts may carry higher levels of DNA damage and *Cdkn1a/*p21^Waf1/Cip1^ expression than NHLFs, but *Cdkn2a*/p16^ink4a^ expression is not a hallmark of commercial IPF cell products. This complicates interrogating fibroblast senescence and fibrosis-related matrix deposition under standard *in vitro* culture conditions given the limited markers. Unbiased, high-throughput approaches such as RNAseq and databases such as the IPF Cell Atlas should be employed for selecting donors to study these complex phenotypes. Our data indicates that vendor-provided primary IPF cell products cannot be assumed to exhibit both canonical senescence and fibrotic markers without a deeper analysis.

### Establishment of a bleomycin-induced senescence model in primary human alveolar epithelial cells (AECs)

The crosstalk between senescent alveolar epithelial cells and lung fibroblasts is an important hallmark of IPF disease. While p16^ink4a^ mRNA-positive epithelial cells are found in the human fibrotic lung (Figure 1) and are hypothesized to be worthy targets of drug discovery efforts [35], there are no commercial sources of diseased human epithelial cells from IPF patients to use for such *in vitro* or *ex vivo* mechanistic studies. This necessitates alternative models to study the interaction of these damaged cells with disease driving fibroblasts.

We used a commercially available product of healthy, primary, human AECs sourced from AcceGen for model development. These adult derived cells are a mix of ATI and ATII cells, and maintain their epithelial phenotype in 2D culture, showing high E-cadherin expression, as well as both ATI and ATII markers through 7 days of culture (Supplementary Figure 3). Moreover, while neonatal AECs tend to transform into mesenchymal-like cells with prolonged culture, these cells show low expression of common mesenchymal genes at days 14 and 21 of growth as a 2D monolayer on transwell inserts (Supplementary Figure 4A). Functionally, these cells continue to form a tight barrier over time, as measured by trans-epithelial electrical resistance (TEER) readings with a transwell system, suggesting they retain their epithelial characteristics for several weeks in culture (Supplementary Figure 4B).

**Figure 3:**
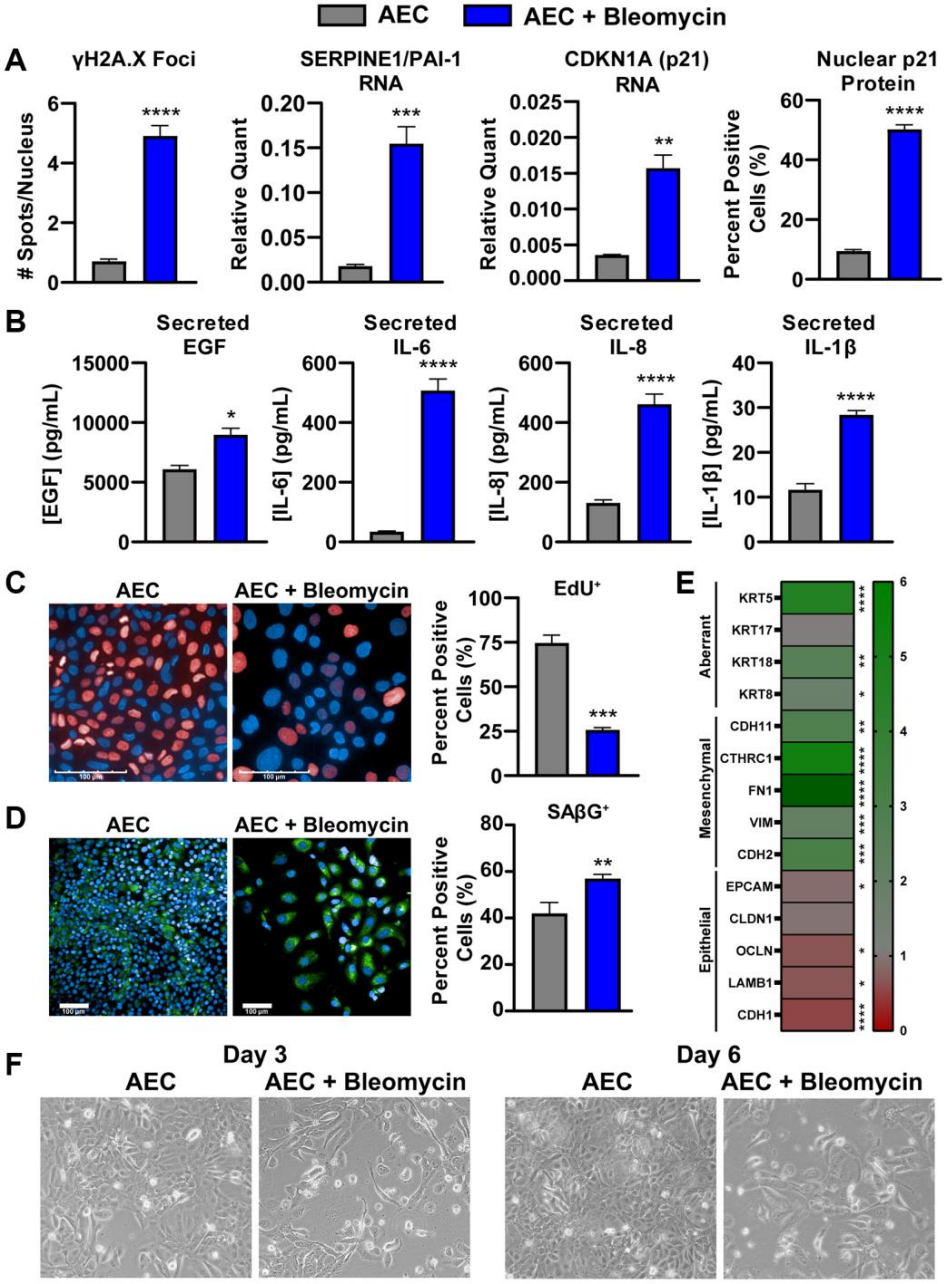
**Bleomycin-damaged primary alveolar epithelial cells exhibit cellular senescence, SASP, and an aberrant phenotype.** (**A**) Following one-week culture post-bleomycin challenge, primary AECs were either fixed for high content immunocytochemistry analysis of nuclear p21 intensity and γH2A.X foci per nucleus or lysed for qPCR gene expression analysis of senescence genes *Cdkn1a* and *Pai1*. For gene expression, data were normalized to *Gapdh* housekeeper expression using the 2^−ΔCt^ method. (**B**) Supernatants from AECs were assayed for components of the SASP (IL-6, IL8, and IL-1β) by MSD kits and EGF by Luminex. (**C**) EdU incorporation was assessed by high content fluorescence analysis in bleomycin-treated and -untreated AECs to show arrest of the cell cycle by determination of Alexa647-positive cells. (**D**) SAβG activity was measured using a fluorogenic probe incubated on fixed cells at pH 6 for and subsequent nuclear staining with Hoechst, high content imaging, and quantitation in Harmony to calculate the percent positive objects. (**E**) AECs were lysed for qPCR gene expression analysis of common epithelial, mesenchymal, or aberrant basaloid markers. Data were normalized to *Gapdh* expression, a fold change value versus untreated AECs was determined using the 2^−ΔΔCt^ method, and fold change data were plotted in a heatmap. (**F**) Representative 10X magnification brightfield images of AECs with and without 24-hr bleomycin treatment at days 3 and 6. Data are representative of three biological donors across 3-5 independent experimental repeats. For all experiments, statistical analysis was performed using unpaired *t*-tests in GraphPad Prism: ^*^p < 0.05, ^**^p < 0.01, ^***^p < 0.001, ^****^p < 0.0001. Error bars represent SEM of *n* = 3 (**A**, **E**), *n* = 3-5 (**B**), or *n* = 2 (**C**, **D**, **F**) technical replicates.

To overcome the lack of commercially available primary, human, diseased AECs from IPF patients, we established a method of *in vitro* bleomycin-induced senescence in these primary AECs to aid in mechanistic studies and pharmacological profiling. Briefly, healthy primary AECs are cultured on a gelatin-based coating solution in microplates with 0.01 U/mL clinical-grade bleomycin to damage the cells for 24 hours. Following a thorough wash, the cells are incubated for an additional week to allow the cells entrance into a growth-arrested, senescent state. These cells show various hallmarks of cellular senescence, notably, increases in γH2A.X foci per nucleus, expression of cell cycle inhibitor *Cdkn1a* (p21) transcript, expression of the p53 target gene transcript *Pai1/Serpin1*, and increases in nuclear p21^Waf1/Cip1^ protein expression (Figure 3A and Supplementary Figure 5A, 5B). Despite a significantly lower final cell density of bleomycin-damaged cells compared to untreated cells due to growth arrest, the senescent cells secrete significantly more IL-6, IL-8, and IL-1β, all components of the inflammatory and damaging SASP (Figure 3B). EGF, a canonical ligand of EGFR, which mediates an important signaling axis for the development of lung fibrosis [36], was also found to be significantly increased in these supernatants (Figure 3B). A newly identified SASP component relevant to IPF, GDF-15 [37], was only increased when concentrations were normalized to account for differences in final cell density (Supplementary Figure 6). Similarly, a multiplex immunoassay was performed on the senescent and non-senescent conditioned media from 3 AEC donors to characterize the secretory phenotype, and many secreted proteins were only significantly increased after normalization to cell density (Supplementary Table 2).

To ensure that these cells are indeed senescent, EdU incorporation and senescence-associated beta-galactosidase (SAβG) activity were measured by fluorescent high content analysis. Significantly fewer EdU-positive cells, suggesting marked growth arrest, and significantly more SAβG-positive cells were identified in the bleomycin-treated conditions (Figure 3C, 3D). Of note, contact-based growth arrest with the higher density of untreated AECs may have resulted in higher background staining of SAβG (Figure 3D), and as such the degree of change with bleomycin treatment may be an underestimate.

While cellular senescence is an established outcome of bleomycin treatment, such a potent chemical-based challenge may elicit a complex, multifaceted phenotype. To that end, we characterized the expression of several lineage genes to determine the phenotype of these damaged AECs. Significant decreases in epithelial genes *Lamb1*, *Cdh1*, and *Ocln* were observed, however only a slight, but significant, decrease in *Epcam* was noted. Conversely, significant increases in mesenchymal genes *Chd2*, *Vim*, *Fn1*, *Cthrc1*, and *Cdh11* were observed, suggesting these cells may transcriptionally shift toward matrix producing cells. Of particular note, bleomycin-treated AECs exhibit significantly higher expression of *Krt8*, *Krt18*, and *Krt5*, which have been recently identified as markers of a transitional, aberrant basaloid population of AECs specifically found in the IPF lung (Figure 3E) [5–8]. Morphological assessment by brightfield imaging on days 3 and 6 post-bleomycin treatment reveals lower cell densities and more spindle-like cells than in untreated conditions, indicative of an aberrant phenotype (Figure 3F). A significant increase in nuclear size, a marker of cellular senescence [38], was also detected by high content analysis (Supplementary Figure 5C). The lower cell density was corroborated by a reduced CellTiter-Glo signal, which reflects ATP levels and can be used as a proxy for the number of viable cells, by the end of the treatment period (Supplementary Figure 5D).

With this phenotype, we believe these bleomycin-damaged AECs represent a highly relevant state of damage-associated transient progenitor epithelial cells found in IPF disease, consistent with recently reported senescence-associated differentiation disorders of pre-alveolar type-1 transitional cell state (PATS) or alveolar differentiation intermediates (ADI) [5–8].

### Direct damage induction of fibroblasts does not induce transcription of matrix components

**Figure 4:**
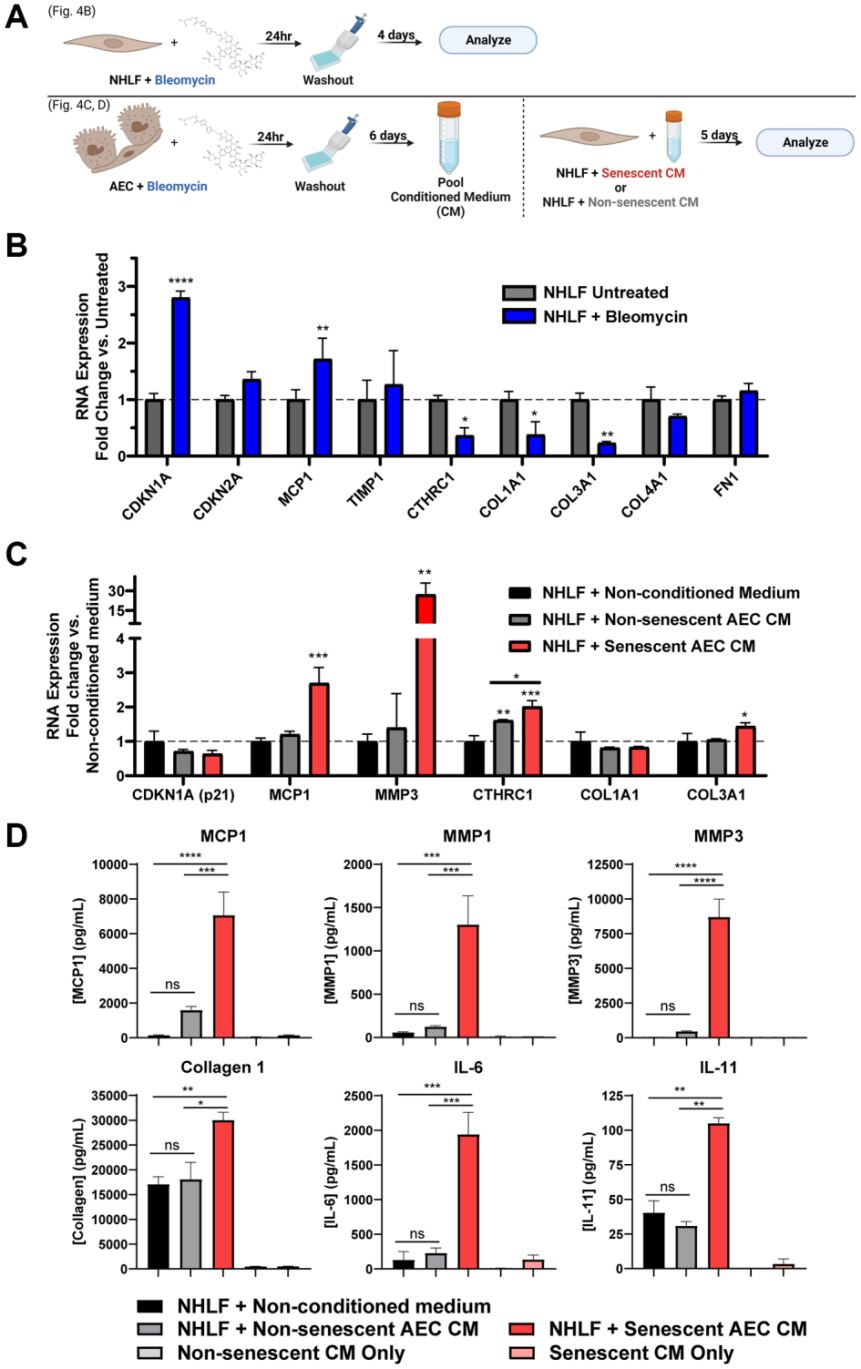
**Secreted factors from senescent alveolar epithelial cells potentiate pro-fibrotic gene expression and protein secretion more robustly than direct damage of fibroblasts.** (**A**) Schematic summarizing the experimental protocol of direct damage of NHLFs with bleomycin or indirect treatment of NHLFs by transferring conditioned medium from bleomycin-treated AECs. Image created with https://www.biorender.com/. (**B**) Treated or untreated NHLF cells were lysed, and qPCR gene expression analysis was performed. Expression of senescence and fibrosis-related matrix and secreted factor genes was assessed, and data were normalized to *β2m* housekeeper expression using the 2^−ΔΔCt^ method. (**C**) Senescent or non-senescent AEC conditioned medium or blank AEC medium was diluted 1:3 in fibroblast medium and incubated on NHLFs for 5 days. Cells were lysed for gene expression analysis of senescence and fibrosis-related genes. (**D**) Supernatants were collected from the treatments described in C and assayed for fibrosis-related secreted proteins by MSD kits. Data are representative of three independent experiments accounting for 3 biological donors of AECs and 2 donors of NHLFs. Statistical analysis was performed using a two-way ANOVA and a Dunnett’s Multiple Comparisons post-test vs. control conditions in GraphPad Prism: ^*^p < 0.05, ^**^p < 0.01, ^***^p < 0.001, ^****^p < 0.0001, ns not significant. Error bars represent SEM of *n* = 2 technical replicates.

It was hypothesized that a chemical-based challenge to damage the NHLFs may induce senescence that coincides with secretion of a pro-fibrotic mix of matrix components and other mediators more so than the standard monoculture of primary IPF fibroblasts without a stimulus (Figure 2). Treating NHLF cells with 0.01 U/mL bleomycin for 24 hours, followed by washing and a 4-day incubation to induce senescence (Figure 4A), resulted in significant increases in *Cdkn1a* and *Mcp1* gene expression. Notably, there were significant decreases in *Col1a1* and *Col3a1* expression, while the expression of other matrix components such as *Col4a1* and *Fn1* remained unaltered (Figure 4B). Additionally, a marker of active, matrix secreting pathogenic fibroblasts, *Cthrc1* [39], was also significantly decreased (Figure 4B). Overall, bleomycin induced senescent or pre-senescent, damaged NHLF cells demonstrate a transcriptional shift away from matrix production.

### Senescent alveolar epithelial cell conditioned medium induces a fibrotic transcriptional phenotype, but not secondary senescence, in NHLFs

In order to model the crosstalk between alveolar epithelial cells and lung fibroblasts in the diseased state, we established a system of conditioned medium transfer from bleomycin-damaged AECs to NHLFs. This conditioned medium (CM) serves as a physiologically relevant stimulus for modeling NHLF activation and function in IPF disease. Briefly, following the AEC damage protocol previously described, the pooled senescent or non-senescent conditioned medium was diluted 1:3 in fibroblast medium and transferred to NHLFs growing in CytoSoft 8 kPa plates with collagen 1 coating for 5 days. Increased expression of fibrosis-relevant genes *Mcp1*, *Mmp3*, and *Col3a1* were observed with senescent, but not non-senescent AEC CM (Figure 4C). Both senescent and non-senescent AEC CM were able to significantly induce expression of the pathogenic fibroblast marker *Cthrc1* compared to the control conditions, and moreover, the senescent AEC CM showed a significant increase compared to the non-senescent AEC CM (Figure 4C). No treatment condition resulted in a significant change in the predominant collagen transcript, *Col1a1* (Figure 4C). Extending the experiment to day eight did not result in SASP-induced secondary senescence evoked by the AEC conditioned medium as evidenced by a lack of *Cdkn1a* and *Cdkn2a* induction (Supplementary Figure 7). These transcriptional readouts suggest that the senescent AEC conditioned medium was capable of inducing a fibrosis-relevant transcriptional profile where direct, senescence-inducing damage to the NHLFs themselves did not.

### Conditioned medium from senescent AECs potentiates a fibrotic secretome in NHLFs

Notably, the senescent, but not non-senescent, AEC CM was able to induce significant secretion of fibrosis-associated proteins MCP1, MMP1, and MMP3, as well as collagen 1 and inflammatory proteins IL-6 and IL-11, compared to the non-conditioned fibroblast medium control condition (Figure 4D). As a control, the AEC CM alone (without NHLF cell incubation) was assayed to ensure that the reported differences in secretion were due to the direct effects of the CM on the fibroblasts. Secretion of TIMP1 and fibronectin were significantly influenced by the use of tissue culture plastic plates and complete growth medium during the experimental period and should be interpreted with caution (Supplementary Figure 8). Crosstalk from senescent AECs is sufficient to elicit a complex NHLF phenotype of ECM catabolism, inflammation, and collagen 1 secretion that resembles a pro-fibrotic disease state in the IPF lung.

### Senolytic treatment of senescent AECs with Navitoclax or AD80 reduces SASP factors and reduces subsequent secretion of collagen by NHLFs

**Figure 5:**
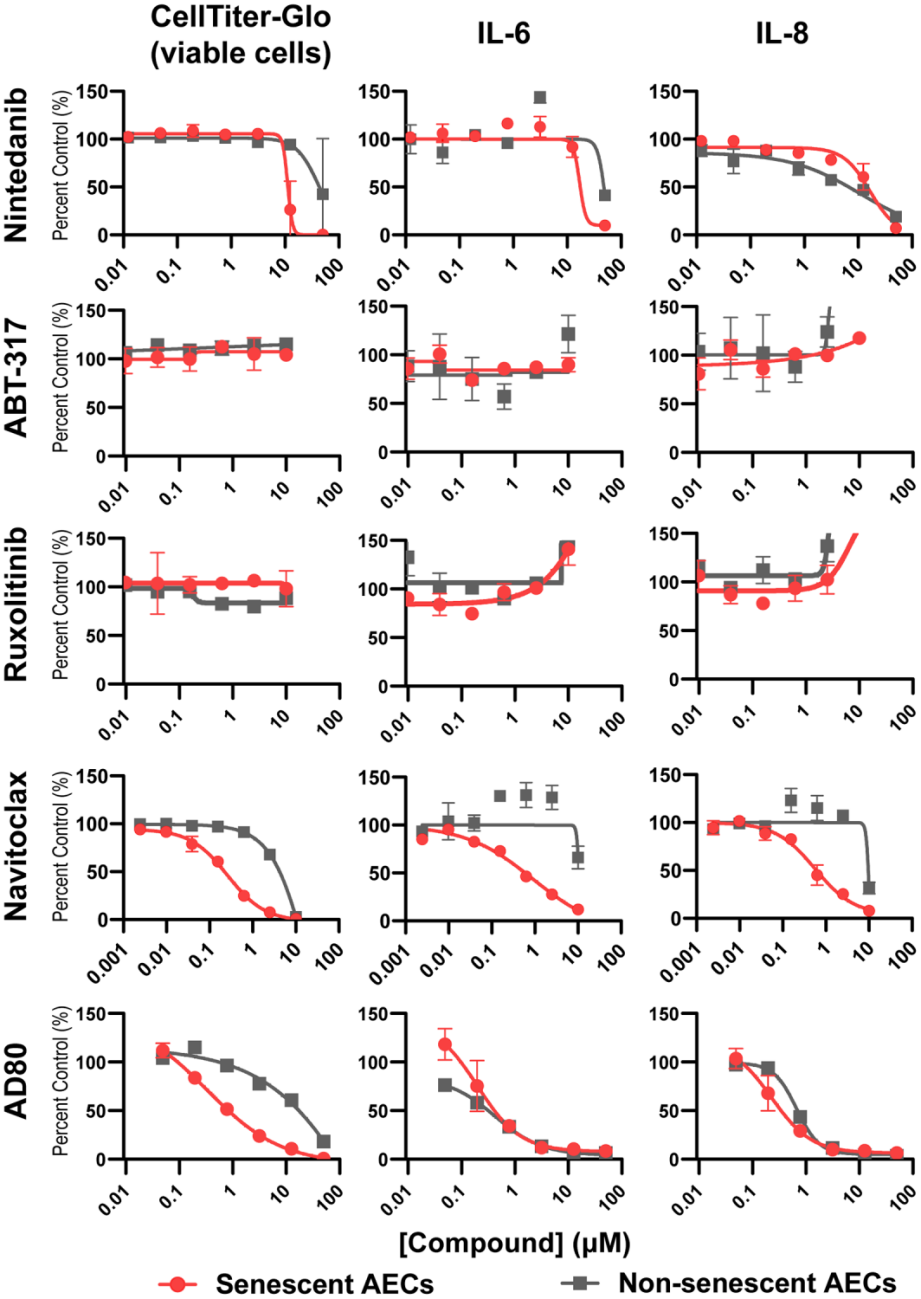
**Senolysis, but not JAK inhibition, reduces key SASP components from senescent AECs.** Four days following medium only or bleomycin treatment of AECs as previously described, medium was refreshed containing DMSO vehicle, or a dose response of Nintedanib, ABT-317, ruxolitinib, Navitoclax, or AD80 for 3 days. Supernatants were assayed for IL-6 and IL-8 using MSD kits. The number of viable cells was assessed with CellTiter-Glo reagent. Curves were fit to a four-parameter logistic function in GraphPad Prism and the IC_50_ values are summarized in Table 1. Representative curves from 3-4 experimental repeats from 3 biological donors are shown. Error bars represent SEM of *n* = 2 technical replicates.

To validate that the model can effectively profile senescence-targeting molecules, tool BCL-2/xL inhibitor Navitoclax, multikinase inhibitor AD80, standard of care drug Nintedanib, and senomorphic JAK inhibitors ABT-317 and ruxolitinib were added to senescent AEC monocultures on day four post-bleomycin treatment for an incubation period of 3 days. As expected, Navitoclax was able to potently and completely kill senescent, but not non-senescent AECs, with a corresponding reduction in the SASP components IL-6 and IL-8. In contrast, Nintedanib showed weak activity, incomplete inhibition, and did not show differential effects on senescent and non-senescent AECs. Senomorphics ABT-317 and ruxolitinib had no effect on the viability of senescent AECs nor did they reduce the SASP IL-6 or IL-8 levels. The multi-kinase inhibitor AD80 showed slight selectivity in killing senescent cells but reduced SASP components with equal potency in senescent and non-senescent AECs, suggesting it acts both as a senomorphic and weakly as a senolytic (Figure 5). Quantitation of IC_50_ and senescence fold selectivity values can be found in Table 1.

**Table 1:** Summary of drugs screened in senescent AEC model, their respective IC_50_s per endpoint, and their fold selectivity for senescent versus non-senescent AECs.

**Drug**	**Mechanism**	**Class**	**IC50 (μM)**						**Senescence Selectivity (fold)**		
			**CellTiter-Glo**		**IL-6**		**IL-8**	
			**Non-senescent AECs**	**Senescent AECs**	**Non-senescent AECs**	**Senescent AECs**	**Non-senescent AECs**	**Senescent AECs**	**CellTiter-Glo**	**IL-6**	**IL-8**
Nintedanib	TGF-β signaling	Anti-fibrotic (SoC)	48.1 ± 3.0	24.1 ± 19.2	50	38.2 ± 14.3	34.2 ± 16.9	37.9 ± 14.4	2.0	1.3	0.9
ABT-317	Jak1	Senomorphic	10	10	10	10	10	10	1.0	1.0	1.0
Ruxolitinib	Jak1/2	Senomorphic	10	10	10	10	10	10	1.0	1.0	1.0
Navitoclax	Bcl-2/xL	Senolytic	2.6 ± 3.8	0.06 ± 0.03	9.4 ± 0.55	0.13 ± 0.03	10	0.07 ± 0.02	44.6	71.0	134.2
AD80	RET, Raf, SRC, S6K	Senomorphic and weak senolytic	2.3 ± 0.5	0.46 ± 0.75	0.5 ± 0.1	0.42 ± 0.13	0.73 ± 0.13	0.37 ± 0.03	5.1	1.2	1.9

Values represent the geometric mean ± SEM of 3-4 experimental repeats.

**Figure 6:**
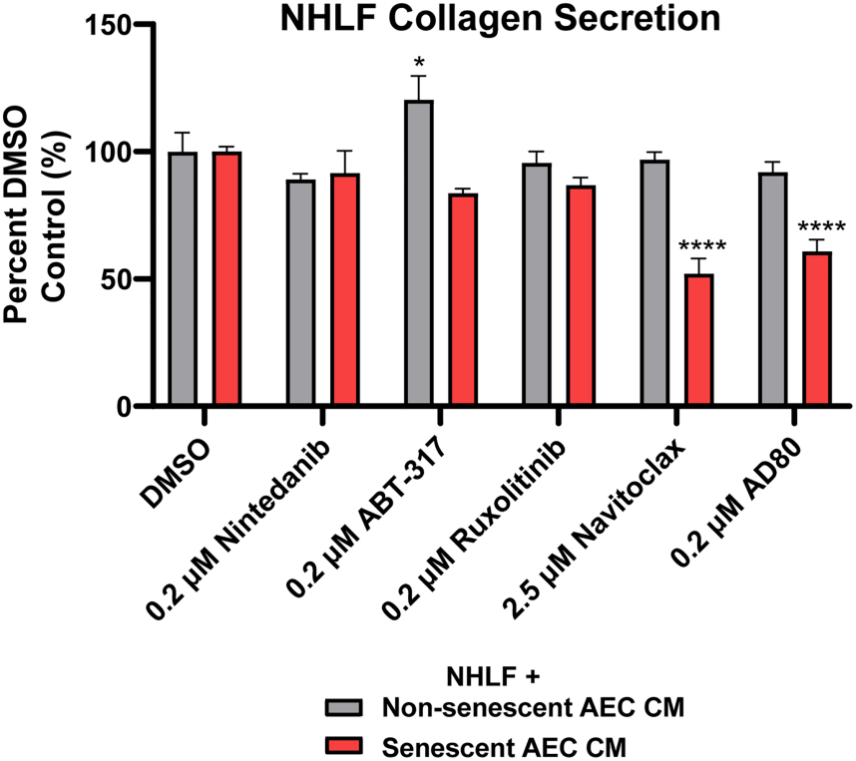
**Senolytic tool agent Navitoclax and AD80 attenuate senescent AEC CM-mediated collagen secretion.** Conditioned medium with 2.5 μM Navitoclax and 200 nM Nintedanib, ABT-317, ruxolitinib, or AD80 AEC treatment conditions from Figure 5 were diluted 1:3 in fibroblast starve medium and transferred to NHLFs for 5 days. Supernatants were assayed for collagen 1 with MSD kits. Data are presented as a percentage of the DMSO control in the matched treatment condition. Statistical analysis was performed using a two-way ANOVA and a Dunnett’s multiple comparisons test in GraphPad Prism versus each group’s DMSO control: ^*^p < 0.05 and ^****^p < 0.0001. Data is representative of three independent experiments accounting for 3 biological donors of AECs and 2 donors of NHLFs. Error bars represent SEM of *n* = 5 (DMSO controls) or *n* = 2 (treatments) technical replicates.

When the AEC conditioned medium following treatment with clinically and physiologically relevant concentrations of Navitoclax (2.5 μM) [40] and Nintedanib (200 nM) [41], and 200 nM of ABT-317, AD80, and ruxolitinib were transferred to NHLFs, senolytic tool Navitoclax and AD80 were able to significantly inhibit the collagen secretion induced by the senescent AECs. Neither Nintedanib nor the senomorphics ABT-317 and ruxolitinib had any inhibitory effects in this system (Figure 6). Furthermore, the inhibition by Navitoclax and AD80 was not due to a direct effect of the drugs on the NHLFs, as shown by the lack of inhibition in non-senescent AEC CM condition (Figure 6). A slight reduction in CellTiter-Glo values was observed on the NHLFs with the Navitoclax and AD80 treatments (Supplementary Figure 9), suggesting some direct cytotoxic effects, however this is unlikely to explain the magnitude of inhibition of collagen secretion (Figure 6). In summary, the ability of this novel, primary cell system to differentiate between the activities of therapeutics with different modalities suggests that it could be tractably applied in IPF drug development.

## DISCUSSION

While large-scale scRNAseq studies have clearly shown upregulation of senescence pathways in certain epithelial cells and *in vivo* models and have implicated senolytics as promising future treatment option for IPF, the *in vitro* and *ex vivo* tools to interrogate these mechanisms are limited. Fibroblasts taken from the lung of IPF patients show signs of damage and preliminary senescence (Figure 2), but are reported to require exogenous stimulation, such as TGF-β, to maintain their *in situ* fibrotic phenotype [42]. The complexity of senescence biology requires IPF-relevant, complex, primary, multi-cellular models to interrogate mechanisms within the niche, but diseased AECs are not commercially available. To this end, we have developed a novel, plate-based system utilizing primary, disease-relevant human cells to model a core epithelial-fibroblast crosstalk paradigm in the pathogenesis of idiopathic pulmonary fibrosis.

**Figure 7:**
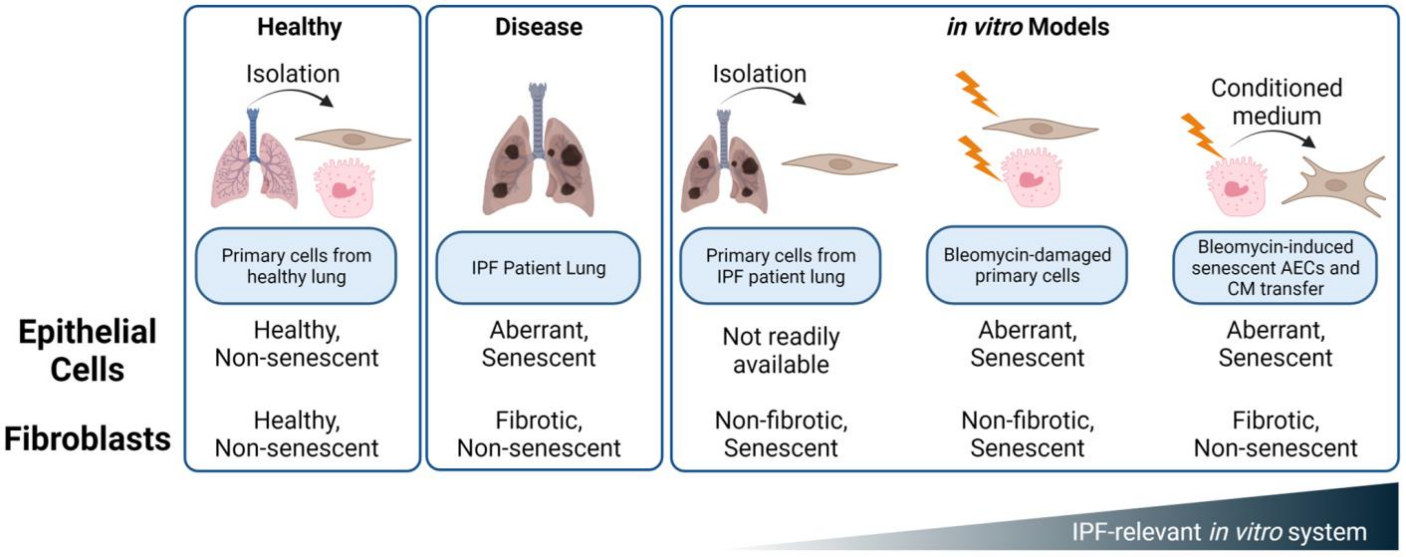
**Summary of *in vitro* and *ex vivo* systems to mimic alveolar epithelial and fibroblast damage and dysfunction in the IPF lung.** While IPF fibroblasts and bleomycin treated NHLFs exhibit some hallmarks of senescence, these cells do not appear to demonstrate a phenotype of *Cdkn2a*/p16^ink4a^ expression, ECM deposition, or secretion of fibrotic mediators such as TIMP1. Rather, the SASP of senescent, aberrant epithelial cells drives a fibrotic phenotype in NHLFs that is consistent with progressive fibrosis. Development of senolytic agents presents an opportunity for therapeutic impact early in disease pathogenesis and with an orthogonal mechanism than the fibroblast targeting standard of care, Nintedanib. Image created with https://www.biorender.com/.

Resident fibroblasts play important roles in tissue homeostasis and maintain a balance between ECM deposition and remodeling. Upon *in vitro* injury and senescence, both intrinsic and stress-induced, fibroblasts switch to an ECM catabolic and inflammatory phenotype characterized by a decrease in ECM component deposition and an increase in MMPs (Figure 4) [43]. We propose that this phenotype is more strongly mediated by the crosstalk between senescent AECs and normal lung fibroblasts than by direct bleomycin damage to the NHLFs themselves. Considering that alveolar epithelial cells are directly injured by environmental damage and stress, and fibroblasts exist in a more protected niche, the model of AEC damage exerting effects on NHLFs reflects the current hypothesis of overall disease initiation and progression (Figure 7).

The striking increase in MMP1 and MMP3 secretion by NHLFs as a result of AEC senescence has not been previously reported (Figure 4). MMP1 has long been regarded as a potential biomarker of fibrotic activity, and has been implicated in the pathogenesis of IPF [44, 45]. In addition to the proteolytic cleavage of both ECM and non-ECM substrates, MMP1 may also promote epithelial cell proliferation and migration [46], thus reinforcing AEC involvement in disease. *Mmp3* RNA and protein expression is correlated to human IPF disease and has been shown to mediate pulmonary fibrosis by activating the β-catenin pathway in lung epithelial cells [47]. Moreover, mice with MMP3 knockout were protected from bleomycin-induced pulmonary fibrosis [47]. Findings from our conditioned medium model present a potential feedback loop wherein damaged AECs potentiate release of these mediators from naïve NHLFs, which may then proceed to damage or further involve naïve AECs and continue the progressive cycle of tissue damage, inflammation, and aberrant repair of the alveolus. Secretion of IL-11, a pleiotropic cytokine and a member of the IL-6 cytokine family, by NHLFs upon senescent AEC condition medium transfer also suggests an autocrine loop and a reinforcement of aberrant epithelial cell differentiation based on previous literature findings with primary IPF cells [48].

The senescence bystander effect has previously been shown with conditioned medium transferred from H_2_O_2_-induced senescent lung fibroblasts to naïve lung fibroblasts, as well as from senescent A549 cells to naïve lung fibroblasts, facilitating an upregulation of SASP component transcripts, p21 protein expression, and DNA damage [49]. Similarly, bleomycin induced senescent AECs and A549s were shown to induce collagen deposition by human embryonic lung fibroblasts via SASP upregulation [14]. Bleomycin-treated A549 cells were previously shown to exhibit mesenchymal like characteristics through activation of the TGF-β/Smad cascades [26]. Conditioned medium from bleomycin-treated MLE-12 cells were shown to induce fibroblast activation and matrix secretion in pulmonary fibroblasts via Wnt/β-catenin signaling [28]. Our model does not appear to capture secondary fibroblast senescence in this timecourse, as senescent AEC CM was unable to induce *Cdkn1a* or *Cdkn2a* expression in NHLFs at days 5 and 8 (Figure 4C and Supplementary Figure 7).

We also confirm and extend the findings of senescent alveolar epithelial cell and fibroblast interaction by characterizing the aberrant and SADD phenotype of bleomycin-treated primary AECs (Figure 3E) and by demonstrating a shift toward ECM catabolism and inflammation that is consistent with fibroblast senescence and IPF phenotypes, despite not observing increases in *Cdkn1a* or *Cdkn2a* in NHLFs themselves following senescent AEC conditioned medium transfer. Fibroblast senescence in fibroblastic foci is correlated to poor outcomes in IPF [50], but the variable expression or lack of expression of p16^ink4a^ in the fibroblastic foci of all IPF patients may suggest that senescence of AECs precedes fibroblast senescence, as AEC senescence has consistently been observed in IPF cells and tissues by our group and others (Figure 1) [14–16, 28]. Overall, this indicates that AECs may be a primary senescent cell type in IPF, and that fibroblast activation is a secondary consequence of AEC growth arrest after damage or injury. A recent study has also pointed to other airway, *e.g.,* bronchiolar, epithelial cells from IPF patients as showing increased senescence gene expression, a SASP, and a bystander effect carried out by extracellular vesicles [51]. As such, the epithelial cell-fibroblast crosstalk mediated by senescent AECs should be directly compared to that of other senescent airway epithelial cells in future studies.

Amelioration of the senescent phenotype of AEC type II cells is an attractive therapeutic hypothesis under exploration for IPF drug development [35, 52]. Disruption of ATII homeostasis through accumulation of senescent ATII cells within the niche not only results in depleted stem cell reservoirs for ATI cell replenishment upon alveolar injury, but also an accumulation of basal cells and a shift toward a pro-fibrotic secretome rather than a regenerative one [13, 35, 53]. Clearance of senescent ATII cells by senolytics or inhibition of the SASP by senomorphic drugs are both strategies under active investigation. Considering the slow accumulation of senescent cells within the environment, it is even hypothesized that such a senolytic therapy could be efficacious with a hit-and-run strategy, allowing patients to go off therapy and continue with other combinatorial drug strategies, thereby reducing safety concerns of taking the therapeutics long-term [54].

We found that senolytic tool compound Navitoclax was able to potently kill senescent AECs, inhibit senescent AEC-derived SASP factors, and reduce collagen secreted by NHLFs upon conditioned medium transfer, while the standard of care agent Nintedanib was not (Figures 5, 6). This data adds additional context to recent findings which suggest that the activity of senescent IPF lung fibroblasts is not impacted by existing standard of care therapies Nintedanib and Pirfenidone [55], as we show that Nintedanib was also unable to attenuate the activity of senescent AECs. The lack of efficacy on senescent cells in the IPF lung may partially explain the limited efficacy of Nintedanib [56], as underlying disease mechanisms may be left unimpacted.

AD80, which showed a slight selectivity for killing senescent AECs at 200 nM, was able to reduce NHLF collagen secretion upon AEC CM transfer (Figures 5, 6, Table 1). p38 gamma and delta are putative targets of AD80 [57], which regulate the SASP via NF-κB [58]. Thus, the marginal senolysis and SASP inhibition this concentration may explain its efficacy in our model. These findings support the hypothesis that next generation senolytics with acceptable safety margins may offer therapeutic benefit over existing standard of care therapies in IPF by acting earlier in disease pathogenesis and curtailing fibroblast activation via elimination of senescent AECs following early damage.

JAK inhibitors ABT-317 and ruxolitinib treatment of senescent AECs failed to prevent IL-6 and IL-8 production in fibroblasts following CM transfer. While JAK inhibitors have been investigated in the context of ILD [59], and are thought to inhibit the SASP [60], it may be that the JAK/STAT pathway is not sufficiently upregulated in senescent AECs and that in this specific context, JAK inhibition is more suited to inhibiting the paracrine effects of the SASP rather than the production of the SASP components themselves. The lack of inhibition of senescent AEC CM-mediated collagen secretion with JAK inhibitors, however, suggests that senomorphics with alternative mechanisms should be investigated to curtail the effects of the senescent AEC SASP.

While our work serves as a proof of concept, we acknowledge several limitations of our current model. The scope of relevant mediators of fibroblast activation presented in Supplementary Table 2 is limited, and large-scale and sensitive metabolome- and proteome-wide characterization of AEC supernatants should be performed to comprehensively profile these mediators. In addition, the consequence of senolysis may release small molecule or protein mediators from dying cells that indirectly impact NHLF activity and should be assessed by alternative detection methods. Additionally, while the lack of efficacy of ABT-317 and ruxolitinib (Figures 5, 6) was unexpected, expression of the JAK/STAT family members should be determined in senescent and non-senescent AECs, as well as the molecular targets of AD80 to assess whether its observed effects may be elicited from off-target activity. Furthermore, disease relevant inducers of senescence can be explored, such as inserting TERT mutations into primary AECs via CRISPR, performing ionizing radiation, inducing replicative senescence, or utilizing hypoxia chambers. This plate-based system is also readily scalable and can be enhanced with a high content screening endpoint in addition to secreted protein analysis to image and quantify dynamics of ECM component deposition or catabolism by the NHLFs that is facilitated by AEC CM transfer, similar to recent “scar in a jar” models [61]. Novel senomorphic and senolytic agents can be evaluated in this model as they are identified. Taken together, the model described herein provides a physiologically relevant, primary human cell system to study the effects of alveolar epithelial cell senescence on lung fibroblasts in the context of chronic fibrotic lung disease.

## MATERIALS AND METHODS

### Cells, and reagents, and *in vitro* procedures

Primary normal human lung fibroblasts (NHLF) were purchased from Lonza and ATCC, and diseased IPF lung fibroblasts were purchased from BioIVT, Lonza, or ATCC. These cells were cultured in fibroblast basal medium supplemented with a low-serum (2%) growth kit (ATCC, Manassas, VA, USA) and 1% penicillin-streptomycin (Thermo Fisher Scientific, Waltham, MA, USA) and expanded to no greater than passage 4. Cells were grown on standard tissue culture plastic flasks for expansion and CytoSoft 8kPa plates (Advanced Biomatrix, Carlsbad, CA, USA) with a VitroCol collagen 1 coating (Advanced Biomatrix) for experiments unless otherwise noted in the figure legend. NHLFs were trypsinized for passage using 0.25% Trypsin-EDTA (Thermo Fisher Scientific). For experiments, NHLFs and IPF fibroblasts were cultured in starve medium, containing 0.5% fetal bovine serum (Thermo Fisher Scientific) in the fibroblast basal medium (ATCC), 1% penicillin-streptomycin, and no additives from the low-serum growth kit.

Primary human alveolar epithelial cells (AECs) were purchased from AcceGen and grown in AcceGen alveolar epithelial basal medium supplemented with a corresponding epithelial growth supplement kit (AcceGen, Fairfield, NJ, USA). All cell culture plates with primary human alveolar epithelial cells were pre-treated with a gelatin-based coating solution (Cell Biologics, Biologics, Chicago, IL, USA) for 2 minutes at room temperature, followed by aspiration but no DPBS washes. AECs were utilized in experiments without expansion and were assayed directly from the vendor-provided cryovials. Unless otherwise noted, all experimental incubations were conducted in a standard cell culture incubator at 37ºC and 5% CO_2_.

Clinical-grade Bleomycin (Meitheal or TEVA) was resuspended at 5U/mL in sterile 0.9% NaCl solution. Aliquots were frozen at −20ºC and were stored for no longer than 6 months. All tool compounds were synthesized by AbbVie, but can be purchased commercially: Navitoclax, Nintedanib, ruxolitinib, AD80 (MedChemExpress, Monmouth Junction, NJ, USA) or ABT-317 (ProbeChem, China). Compounds were stored solubilized at 10mM in 100% dimethyl sulfoxide (DMSO) (Sigma, St. Louis, MO, USA). Aliquots of 10mM compound stocks were stored long-term in a −20ºC Liconic freezer and storage system. The final concentration of DMSO on cells was no greater than 0.5%.

### RNA *in situ* hybridization of human lung samples

Lung tissue from patients diagnosed with Idiopathic Pulmonary Fibrosis (IPF) or Interstitial Lung Disease (ILD) were obtained from National Disease Research Interchange (NDRI), and histologic assessment confirmed temporal heterogeneity with the presence of both active fibroblastic foci and chronic honeycomb pattern, consistent with the diagnosis of IPF or ILD. All blocks were sectioned at 5 μm in DEPC-treated water. *In situ* hybridization was performed on the Leica Bond RX automated immunostainer (Leica Biosystems, Deer Park, IL, USA) using Advanced Cell Diagnostic’s (ACD/Bio-Techne, Newark, CA, USA) BaseScope LS reagent assay (cat. no. 323600). ACD’s BaseScope assay incorporates a series of proprietary reagents; epitope retrieval was done with both Leica’s Epitope Retrieval 2 (cat. no. AR9640) and ACD’s protease. ACD’s human CDKN2A-2zz-st probe (cat. no. 707578) was run; a positive control probe, Hs-PPIB-3zz (cat. no. 701038), and a negative control probe, dapB-3zz (cat. no. 701018), were also run to ensure the assay was working properly. All blocks were screened with house-keeping gene *Ppib*, prior to running with human *Cdkn2a*, to ensure that they had acceptable mRNA integrity. Visualization was performed using ACD’s BaseScope assay in conjunction with Leica’s Refine Red Detection kit (cat. no. DS9390). The slides were scanned on a Pannoramic 250 whole slide digital scanner (3DHISTECH Ltd, Budapest, Hungary) using a 40X lens with extended focus and the amount and localization of ISH signal was evaluated by a pathologist using a semi-quantitative 0–3 scale (0 = none present, 1 = low signal in a few cells, 2 = moderate signal in a moderate number of cells, 3 = strong signal in numerous cells).

### Induction of senescence in primary human alveolar epithelial cells and conditioned medium preparation

Primary human alveolar epithelial cells are seeded on 12- or 96-well flat bottom tissue culture plates (Corning Inc., Corning, NY, USA) pre-coated with a gelatin-based coating solution (Cell Biologics) and allowed to attach and grow until 25% confluency. Growth medium is then replaced with or without final 0.01 U/mL clinical-grade bleomycin for 24 hours to induce damage and cellular senescence. Cells are thoroughly washed in growth medium and fresh media is left on the cells for one week to establish growth arrest, with medium renewal on day four. If included, addition of inhibitors took place on day four post-washout, and cells were incubated for an additional 3 days before analyzing supernatants or cells. Supernatants are assayed for common SASP components as described below. Cells are lysed for RNA and gene expression profiling or fixed for high content imaging analysis (both described below) to corroborate senescent phenotype. Pooled conditioned medium is aliquoted and frozen at −80ºC for long-term storage and multiple freeze/thaw cycles are avoided.

### Conditioned medium NHLF model

Normal Human Lung Fibroblasts (NHLFs) are allowed to attach overnight in 24- or 96-well CytoSoft 8kPa plates with collagen 1 coating. Senescent or non-senescent alveolar epithelial cell conditioned medium, prepared as described above, is diluted 1:3 in fibroblast starve medium and added to the NHLFs for 5 days. Non-conditioned alveolar epithelial cell medium is also diluted 1:3 in non-conditioned fibroblast medium as a discrete control to account for differences in basal medium composition. Medium and treatments are not renewed during the 5-day incubation period. Upon the completion of the study, supernatants are pulled for secreted protein analyses, and cells are either lysed for RNA and associated gene expression profiling or fixed for high content imaging analysis (both described below). For NHLF studies following treatment of AECs with inhibitors, CM was transferred from the AEC microplate as described above without a freeze/thaw.

### Gene expression studies

Cell samples were DPBS-washed and lysed in 600 μL RLT buffer (Qiagen, The Netherlands) supplemented with 10 μl/mL 2-mercaptoethanol (Thermo Fisher Scientific) and frozen at −80ºC. Total RNA was prepared by a QIAcube automated instrument and an RNeasy isolation kit (Qiagen), and a quality control check was conducted using a QIAxpert UV/VIS spectrophotometer system (Qiagen). Total RNA was converted to cDNA using a high-capacity cDNA conversion kit (Applied Biosystems, Waltham, MA, USA) according to the manufacturer’s instructions. Human gene expression profiling was executed using TaqMan Universal 2X Master Mix (Applied Biosystems) and the probes listed in Supplementary Table 3. Samples were run with technical duplicate. All quantitative real-time PCR experiments were conducted using a ViiA 7 or Quant Studio 12K Flex instrument (both Applied Biosystems). Data was interpreted using a ΔCt normalization method to a housekeeper gene listed in the figure legend to determine relative expression (2^−ΔCt^) or as a fold change versus a control group (2^−ΔΔCt^), as noted in the figure legend.

### Immunocytochemistry and high content imaging

For bleomycin-treatment experiments, cells were fixed in the experimental culture plate by washing with DPBS and incubating with final 4% paraformaldehyde (Thermo Fisher Scientific) diluted in DPBS for 20 minutes at room temperature. Cells were then permeabilized with 0.2% Triton X-100 (Sigma) in DPBS for 2 minutes at room temperature, followed by thorough washing with DPBS. Samples were blocked for one hour at room temperature using final 1% BSA in PBS reagent (Thermo Fisher Scientific). Without washing, primary antibodies against p21^Waf1/Cip1^ (Cell Signaling Technologies, Danvers, MA, USA) or γH2A.X (S139) (Cell Signaling Technologies) were diluted according to manufacturer’s recommendations in blocking buffer and were added to cells overnight at 4ºC. Cells were washed with DPBS and incubated with an Alexa555- or Alexa647-conjugated secondary reagent consisting of the F(ab’)_2_ IgG fragment (Cell Signaling Technologies) diluted at 1:1500 in blocking buffer for 45 minutes at room temperature, protected from light. Following washing, 10mg/mL stock Hoechst 33342 (Thermo Fisher Scientific) was diluted 1:10000 in DPBS and added to the cells for 5 minutes at room temperature, protected from light. Cells were washed a final time in DPBS and imaged on an Operetta High Content Screening instrument (PerkinElmer, Waltham, MA, USA). Representative images were taken of each well at 40X magnification using a water objective, and fluorescent intensities of p21 were calculated within nuclear objects identified by the Hoechst stain. To determine percent positive cells, a threshold was drawn on the distribution of objects based on control, untreated conditions such that objects with greater fluorescent intensities were reported as positive, and lesser intensities reported as negative. γH2A.X foci were identified using a spot analysis in the Harmony software (Version 8, PerkinElmer), and the number of foci per Hoechst-positive object was determined. Objects with greater than one γH2A.X focus were reported as a positive cell.

For AEC characterization studies, cells were fixed with 2% PFA for 10 minutes, washed, and permeabilized with 0.1% Triton X-100 for 10 minutes, followed by thorough washing with DPBS. Cells were blocked for 30 minutes in 5% normal goat serum (Thermo Fisher Scientific) at room temperature. Without washing, primary antibodies against E-cadherin (Cell Signaling Technologies), ATI 56 (Terrace Biotech, San Francisco, CA, USA), or ATII 280 (Terrace Biotech) were diluted according to manufacturer’s recommendations and added to cells for one hour at room temperature. Cells were washed with DPS and incubated with an anti-rabbit Alexa594 (Cell Signaling Technologies) or anti-mouse Alexa488 (Cell Signaling Technologies) secondary reagent, both diluted 1:200, for one hour at room temperature, protected from light. Cells were washed, treated with Hoechst 33342 as described above, and imaged on an Operetta High Content Screening instrument with a 20X objective. Fluorescence intensities were quantitated using Harmony and presented as a percentage of high expressing cells.

To quantify nuclear area of AECs, objects were defined by the Hoechst channel and Harmony software was used to calculate the average nuclear area per well in μm^2^.

### EdU high content analysis

Following bleomycin-induced senescence, AECs are grown in standard growth medium supplemented with 10 μM EdU (Thermo Fisher Scientific) for 24 hours. Cells are subsequently fixed with 4% paraformaldehyde for 15 minutes, washed, and permeabilized with 0.5% Triton X-100 for 20 minutes at room temperature. EdU was detected using the Click-iT EdU Alexa Fluor 647 Imaging kit (Thermo Fisher Scientific) according to manufacturer's instructions. Plates were then imaged on an Operetta High Content Screening instrument at 40X magnification to determine the percentage of Hoechst-positive objects that were also AF647-positive using Harmony software.

### Senescence-associated beta-galactosidase (SAβG) analysis

Following bleomycin-induced senescence, cells are treated in the experimental plate with 100nM Bafilomycin A1 (Invivogen, San Diego, CA, USA) in growth medium for 45 minutes at 37ºC and 5% CO_2_ to stabilize lysosomal pH and reduce false positive background stain. Cells are subsequently fixed with 4% PFA, washed, and the CellEvent Senescence Green beta-galactosidase probe (Thermo Fisher Scientific) is prepared in the supplied buffer at pH 6.0 as described in the package insert. The plate is incubated at 37ºC without CO_2_ for two hours, followed by washing and a Hoechst stain as previously described. The plate is washed a final time with DPBS before being read on the Operetta High Content Screening instrument with Hoechst and FITC channels. Percent SAβG positive cells are calculated using a threshold setting in Harmony software.

### Secreted protein analyses by MSD

Cell culture supernatants were assayed for human cytokines, fibrosis-related proteins, and ECM components using Meso Scale Discovery U-PLEX (MCP1), R-PLEX (TIMP1, IL-11, IL-6, Fibronectin, GDF-15, Pro-Collagen 1, MMP1, MMP3) or Ultra-sensitive Tissue Culture (MMP1,3,9 multiplex) kits according to the manufacturer’s protocols. Standard curves were fit to a 4-parameter logistical function using XCelfit (IDBS, version 5.0). Samples were appropriately diluted to ensure interpolation from the standard curve was based on the linear range of detection. Repeat assays on supernatants were performed with no more than two freeze/thaw cycles.

### Secreted protein analyses by Luminex

Following the senescence induction protocol for AECs, supernatants from three biological replicates were assayed neat with a Milliplex 47-plex Human Cytokine/Chemokine/Growth Factor A kit (Millipore, Burlington, MA, USA) according to the manufacturer’s instructions. IL-1β, IL-6, and IL-8 were omitted from this kit as they are separately measured by MSD. Concentrations were interpolated using a four-parameter logistic function in BioPlex software (version 5.0). To account for differences in cell density, data are presented both as raw concentrations, as well as normalized to the CellTiter-Glo counts (method below) to generate a ratio. This ratio was compared as a fold change in bleomycin-treated AECs versus untreated AECs and a paired *t*-test was performed in Prism.

### Viability analysis by CellTiter-Glo

Following removal of supernatants from experimental plates, 50 μL of assay medium was replaced and 50 μL reconstituted reagent from the CellTiter-Glo Luminescent Cell Viability Assay kit (Promega, Madison, WI, USA) was added per well. The plate was incubated with orbital shaking for 30 minutes at room temperature, protected from light. If black, clear, or clear-bottom plates were used, contents were transferred to opaque white plates (Corning), protected from light. Luminescent measurement was conducted on an Envision microplate reader (PerkinElmer).

### Transwell culture and TEER measurement

AECs were seeded at a density of 40,000 cells/well on PET transwells (Corning) in a 24-well plate (Collagen) format coated with VitroCol collagen 1 and fibronectin (Advanced Biomatrix) in full growth medium. TEER measurements were taken over a timecourse with a chopstick electrode connected to a standard voltmeter and data in Ohms were plotted over time.

### Statistical analysis

For studies consisting of two groups, an unpaired *t*-test was performed. For studies with three or more groups or with multiple variables, an ANOVA assessment was performed with a Dunnett’s, Bonferroni, or a Šídák’s post-test. Any multiple comparisons post-test versus a control group are noted in the figure legends. For serial dose responses, curves were fit to a four-parameter logistic function. All statistical calculations were performed in GraphPad Prism software (GraphPad, version 9.3.1) unless otherwise noted in the figure legend.

## Supplementary Materials

Supplementary Figures

Supplementary Tables
