# Supplementary material for: Modulating in vitro lung fibroblast activation via senolysis of senescent human alveolar epithelial cells
Source: Aging (Albany NY). 2024 Jun 29;16(13):10694–723. doi: 10.18632/aging.205994 (PMC11272128; doi:10.18632/aging.205994)
Supplement: Supplementary Figures [file aging-16-205994-s001.pdf]

SUPPLEMENTARY FIGURES

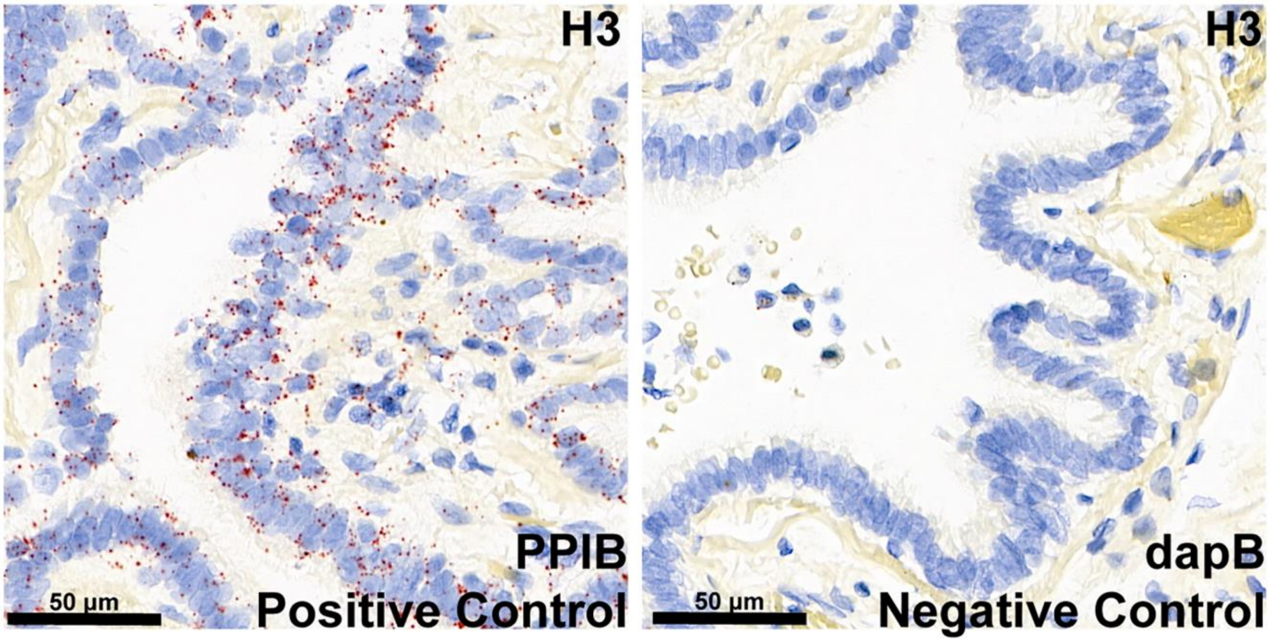

Supplementary Figure 1. Control ISH probes for PPIB and dapB confirm high RNA quality of human lung histology slides across cell types.

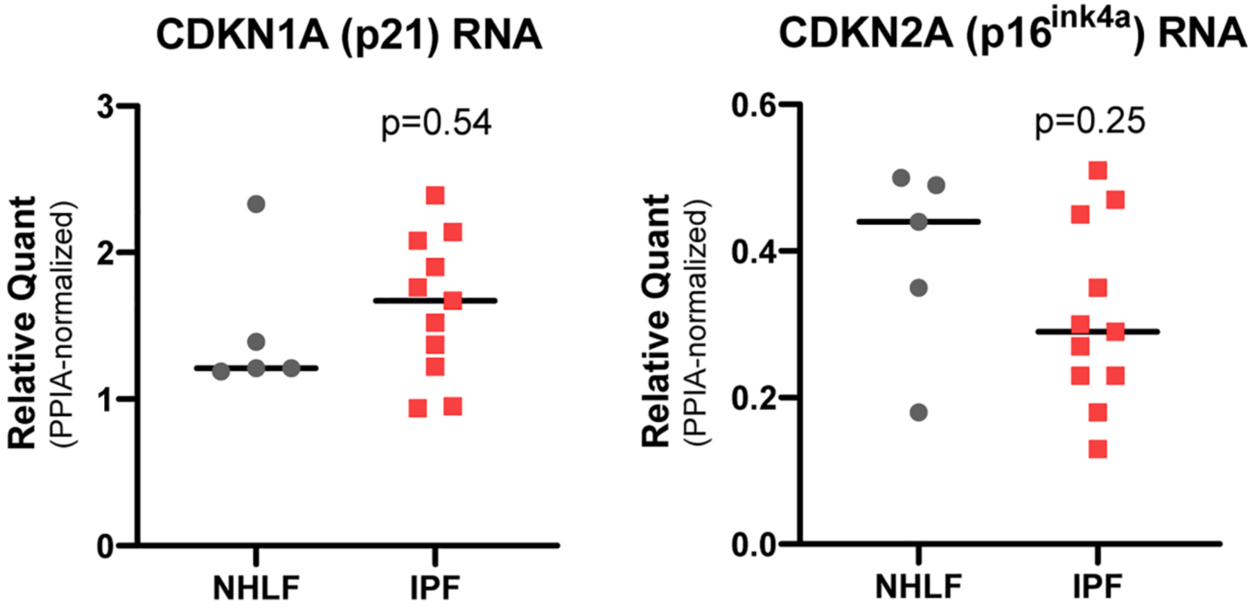

Supplementary Figure 2. Primary lung fibroblasts across vendors show variable expression of core senescence markers *Cdkn1a* (p21) and *Cdkn2a* (p16^{ink4a}). *n* = 11 IPF and *n* = 5 NHLF primary donor cells were lysed immediately after thawing from vendor-provided cryovials. RNA was isolated and gene expression was determined via TaqMan chemistry and normalization to housekeeper gene *Ppia* using the $2^{-\Delta Ct}$ method. Statistical analysis was performed by an unpaired *t*-test and the resulting *p*-value is noted on the figure. Bar represents the median datapoint.

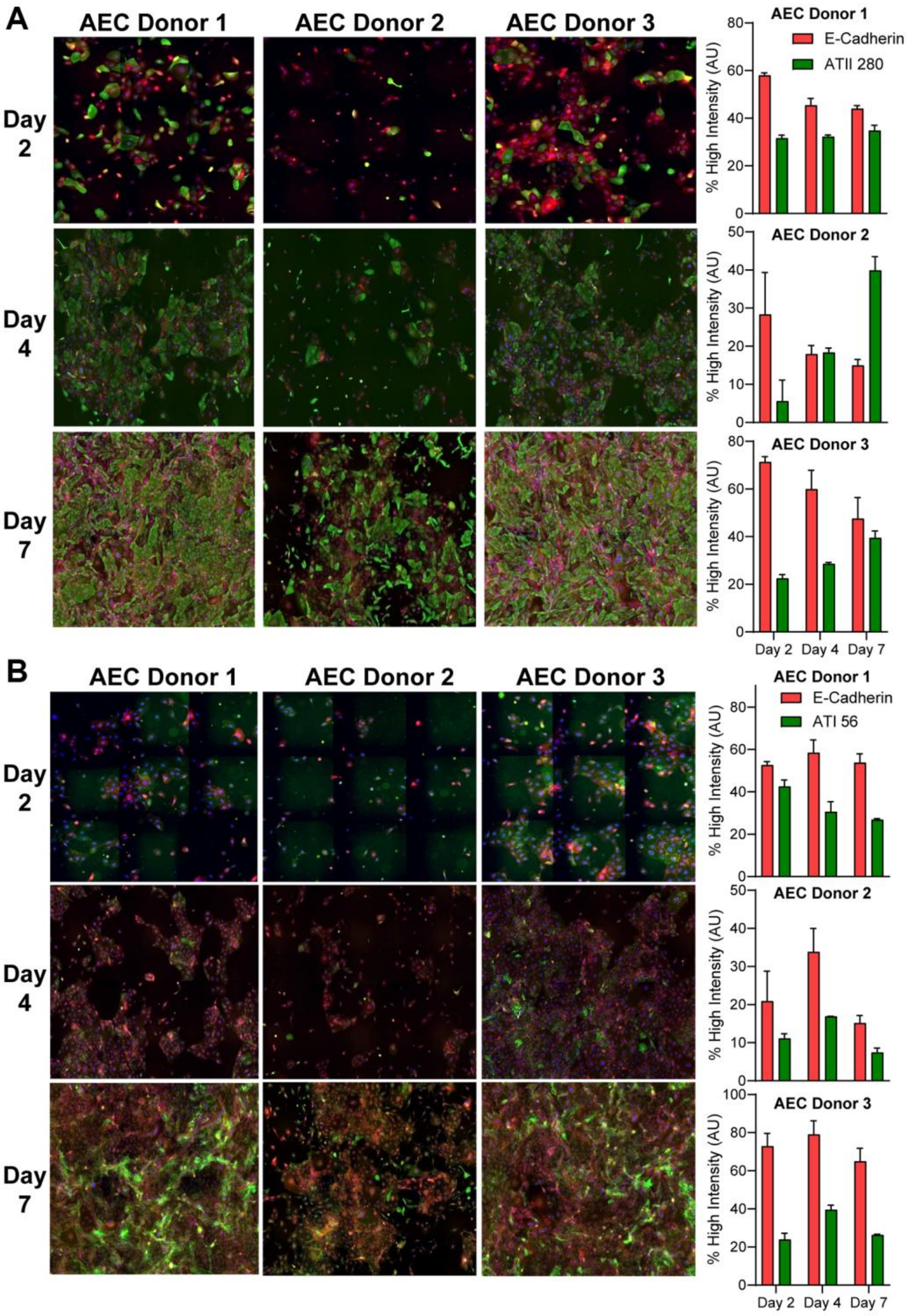

Supplementary Figure 3. Primary AECs maintain morphology, E-cadherin expression, and ATI/ATII marker expression through 7 days of *in vitro* 2D culture. Primary AECs (n = 3 different donors) were thawed from the vendor-provided cryovial and plated in tissue culture plates in full growth medium for 2, 4, or 7 days prior to high content imaging for E-cadherin (red) and (**A**) ATII 280 (green) or (**B**) E-cadherin and ATI 56 (green). Representative 20X images are shown. Images were quantitated using Harmony and the percentage of high fluorescence intensity cells is presented. Error bar represents standard error of the mean (SEM) of n = 2 technical replicates.

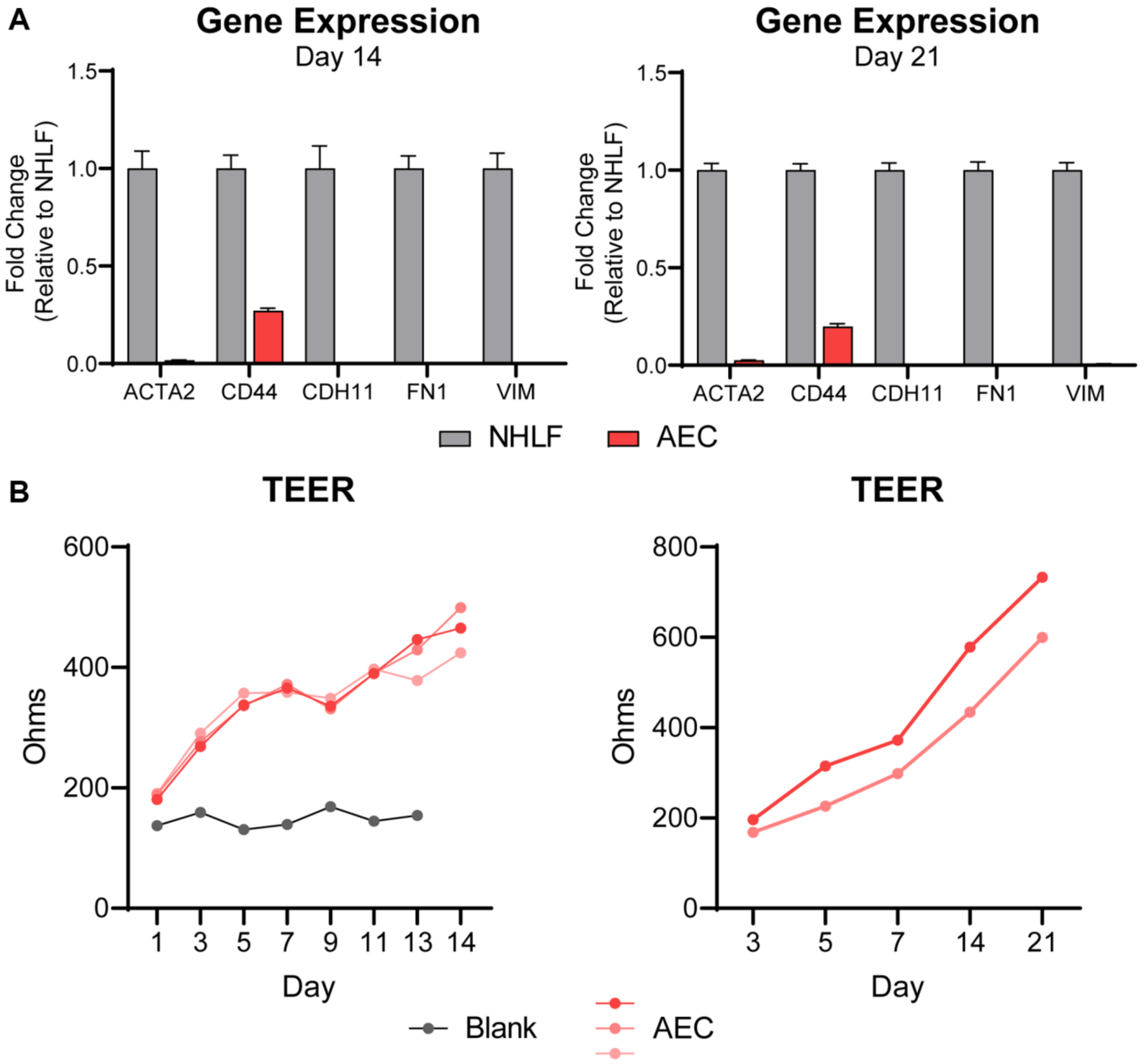

Supplementary Figure 4. Primary AECs do not express mesenchymal gene expression through 21 days of *in vitro* transwell culture and maintain the ability to form a tight barrier. (**A**) Gene expression profiling via TaqMan qPCR of mesenchymal markers was performed at days 14 or 21 of *in vitro* transwell culture where primary AECs were seeded on the apical side. Data were normalized to NHLFs drown on standard 2D culture plates using the $2^{-\Delta\Delta Ct}$ method. (**B**) TEER resistance measurements over time from AECs growing on transwells suggest AECs maintain their ability to form tight barriers through day 21. Resistance of blank transwell with no cells over time shown as negative control (left). Technical well-level replicates are represented by each line. Data shown represent two of three independent experiments accounting for 2 AEC donors and 1 NHLF donor. Error bars represent SEM of *n* = 2 technical replicates.

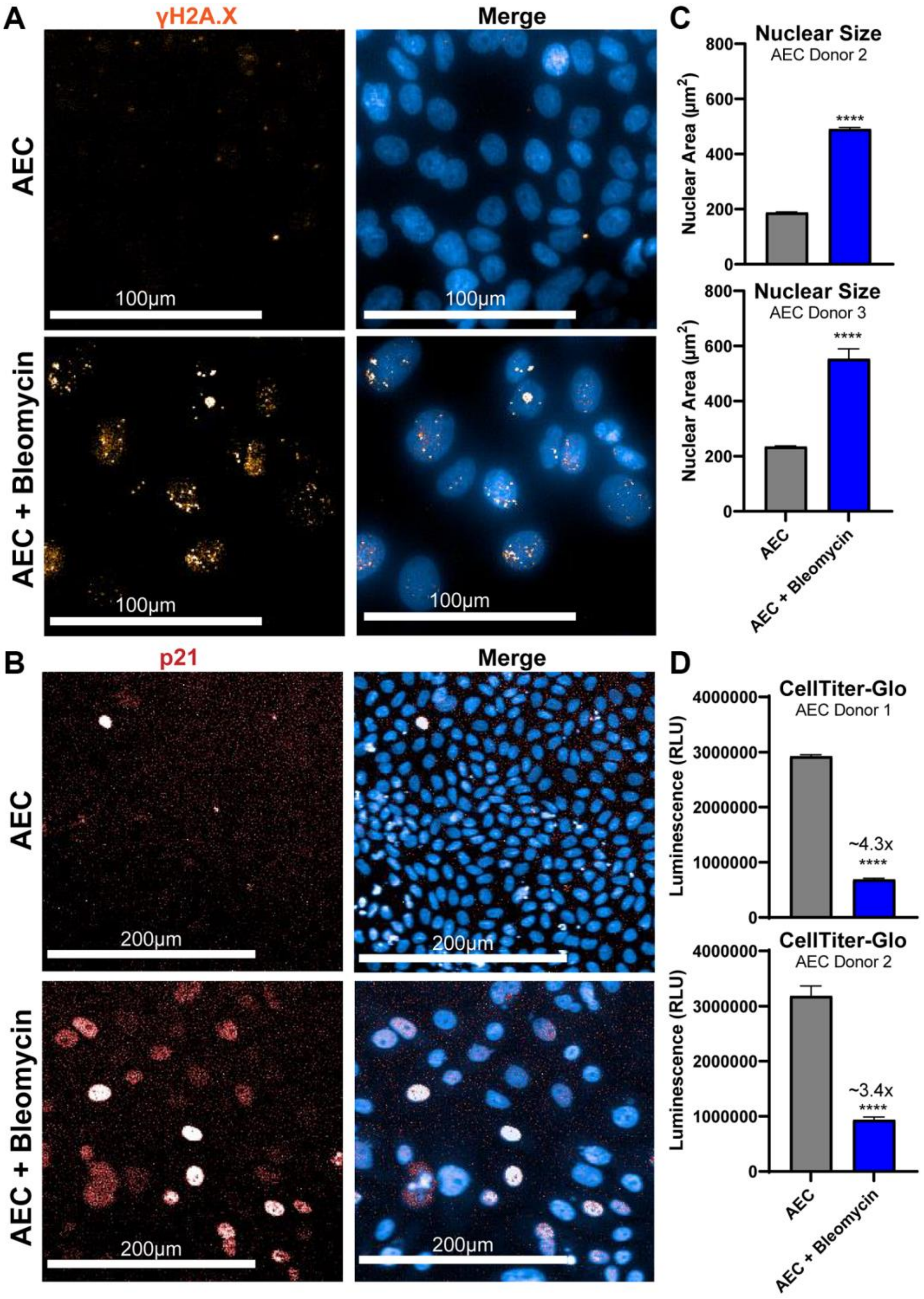

Supplementary Figure 5. Additional characterization of bleomycin-treated AEC cultures. 24-hour bleomycin treatment of AECs results in increased γH2A.X foci per nucleus (**A**) and increases in nuclear p21 protein expression (**B**) after 6 days in culture. Images from these studies were used for the quantitation in Figure 3A. Scale bars are noted on each image. Representative images reflect n = 3 biological AEC donor replicates across 3 independent experiments. (**C**) Nuclear area using the Hoechst channel was determined by Harmony software following Operetta High Content Imaging. (**D**) To estimate differences in viable cells following the study, CellTiter-Glo measurements were taken of control wells and raw luminescence values are plotted. For C and D, two of three independent experiments and AEC donors are shown. $^{****}p < 0.0001$ by paired t-test in GraphPad Prism. Error bars represent SEM of n = 2 (**A**, **B**) or n = 5 (**C**, **D**) technical replicates.

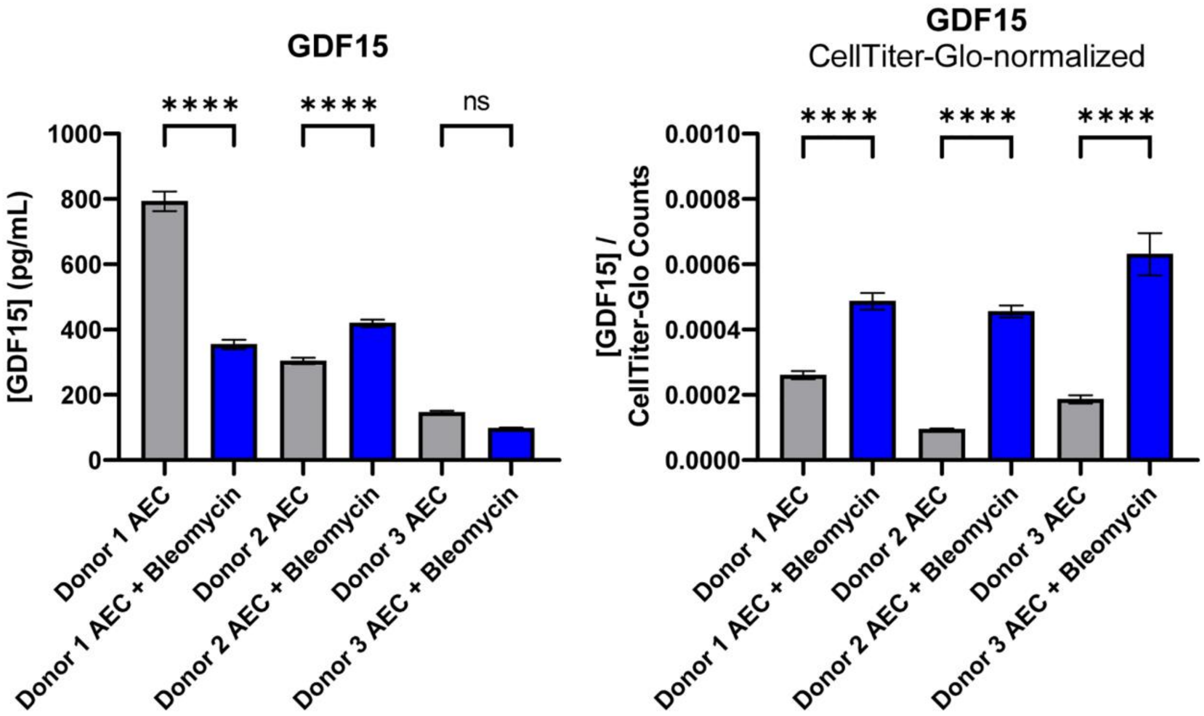

Supplementary Figure 6. GDF-15 concentrations in bleomycin-treated and untreated AEC cultures. Concentrations were determined by MSD on day 6. To account for differences in viable cells at the end of the model, raw concentrations were normalized to CellTiter-Glo counts and the ratio was plotted. Three AEC donors are plotted, representing 3 independent experiments. $^{****}p < 0.0001$ by paired *t*-test in GraphPad Prism. Error bars represent SEM of $n = 5$ technical replicates per donor.

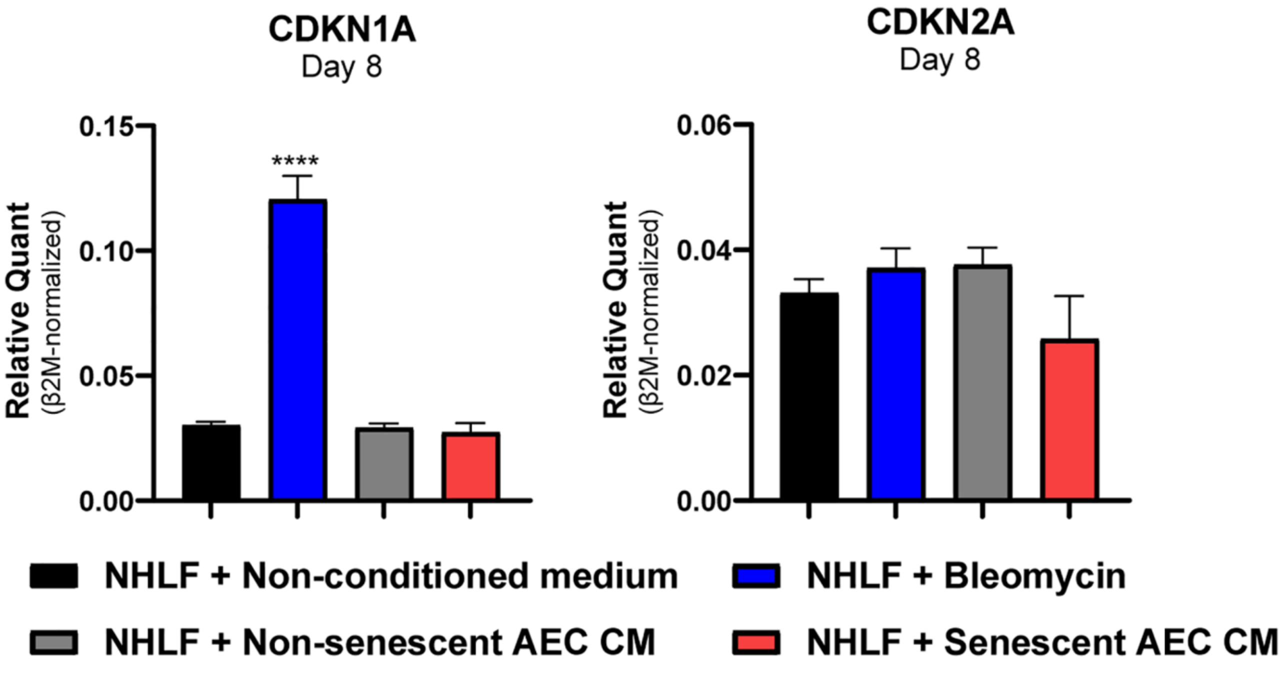

Supplementary Figure 7. Senescent alveolar epithelial cell conditioned medium does not induce secondary senescence of NHLFs by day 8. Following the experimental protocol from Figure 4B extended to a longer NHLF treatment period does not show increased expression of senescence markers *Cdkn1a* or *Cdkn2a* by qPCR. Data were normalized to expression of housekeeper gene *β2m* using the $2^{-\Delta Ct}$ method. Statistical analysis was performed by one-way ANOVA and Dunnett's multiple comparisons post-test vs. the NHLF + non-conditioned medium group: $^{****}p < 0.0001$. Data are representative of 2 experimental repeats. Error bars represent SEM of $n = 2$ technical replicates.

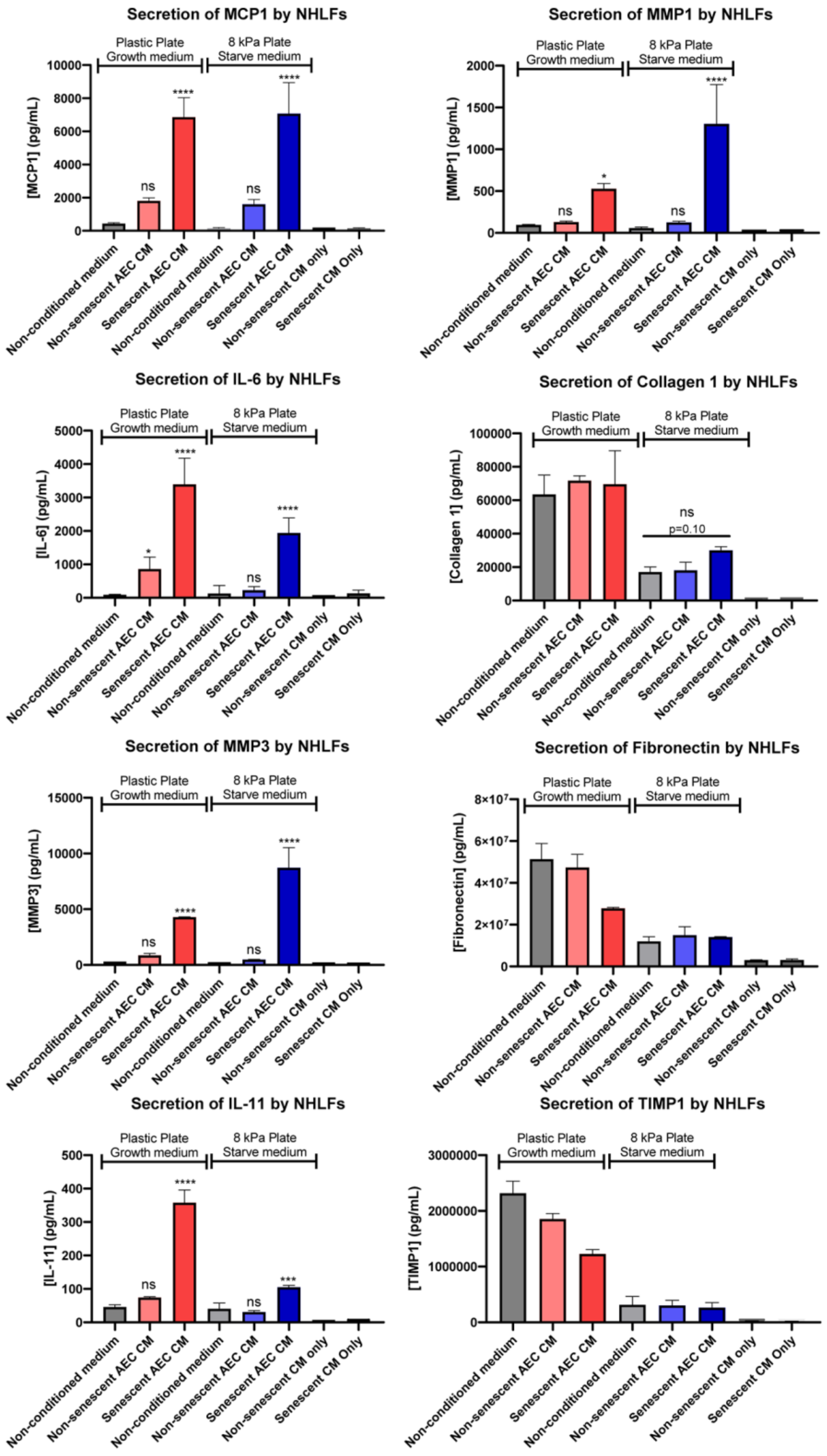

Supplementary Figure 8. Effects of plate type and media conditions on secretion of inflammatory and fibrotic protein secretion by NHLFs. Cells were incubated with indicated treatments on either standard tissue culture plastic plates and full growth medium (2% serum + fibroblast growth factors provided in supplement kit) or 8 kPa CytoSoft plates with collagen 1 coating and starve medium (0.5% serum and no fibroblast growth factors) for 5 days. Secretion of fibronectin, collagen, and TIMP1 was considered to be influenced by culture conditions. Statistical analysis was performed by a one-way ANOVA and a Šídák's multiple comparisons test vs. the non-conditioned medium within each plate condition. $^{*}p < 0.05$, $^{**}p < 0.01$, $^{***}p < 0.001$, $^{****}p < 0.0001$, ns not significant. Data are representative of two independent studies. Error bars represent SEM of $n = 3$ technical replicates.

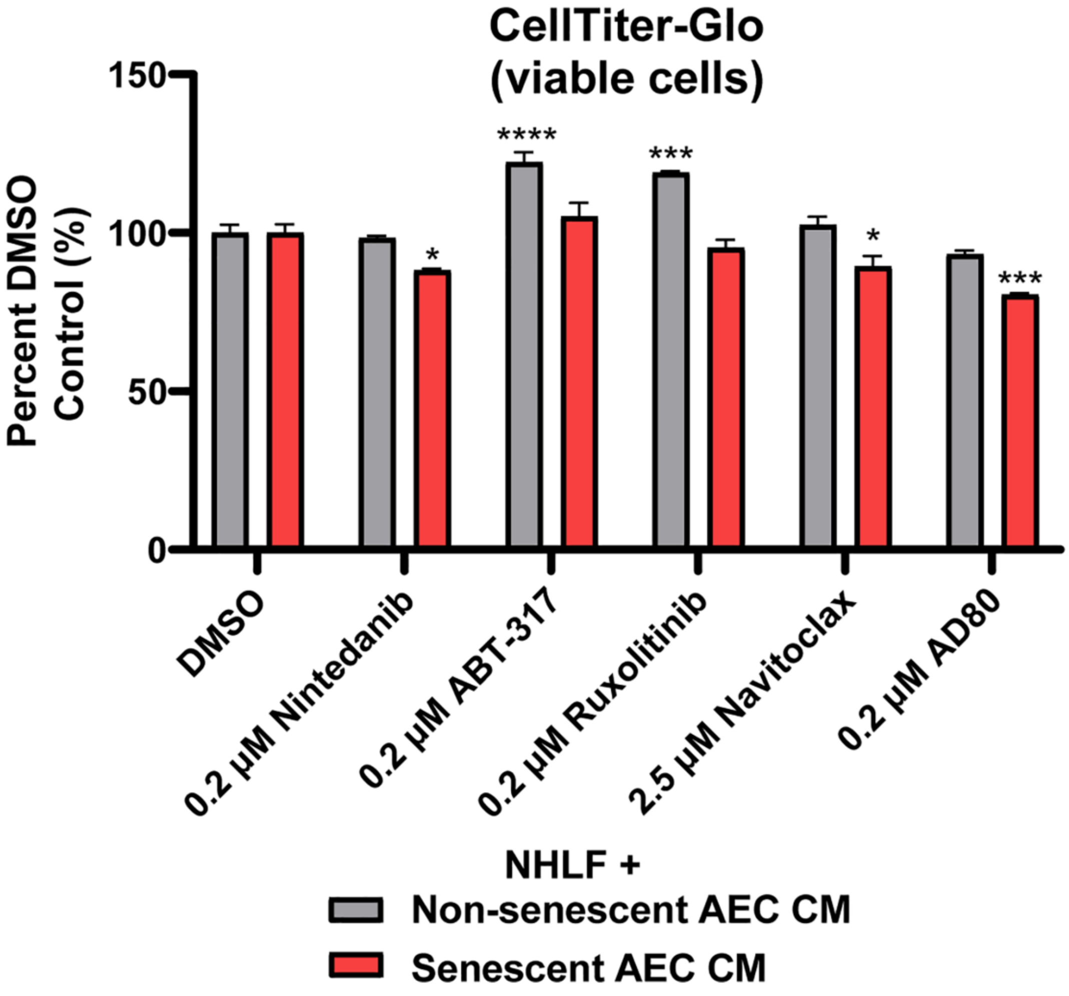

Supplementary Figure 9. Effects of compound-treated AEC CM on NHLF viability by CellTiter-Glo. Following 5 days of treatment as in Figure 6 and supernatant removal, NHLFs were subjected to CellTiter-Glo analysis to account for loss in viable cells. Data are plotted as a percent of DMSO control wells within the same condition. Two-way ANOVA and Dunnett's multiple comparisons test were performed inGraphPad Prism: $^{*}p < 0.05$, $^{***}p < 0.001$, $^{****}p < 0.0001$. Data are representative of three independent experiments. Error bars represent SEM of $n = 5$ wells (DMSO controls) or $n = 2$ wells (treatments).
