# Supplementary material for: Modulating in vitro lung fibroblast activation via senolysis of senescent human alveolar epithelial cells
Source: Aging (Albany NY). 2024 Jun 29;16(13):10694–723. doi: 10.18632/aging.205994 (PMC11272128; doi:10.18632/aging.205994)
Supplement: Supplementary Tables [file aging-16-205994-s002.pdf]

SUPPLEMENTARY TABLES

Supplementary Table 1. Demographic information of human lung tissue donors.

Identifier	Condition	Age	Sex	Ethnicity
H1	Non-fibrotic Lung	57 yr	Male	Caucasian
H2	IPF Lung	57 yr	Male	Hispanic
H3	IPF Lung	72 yr	Male	Not disclosed
H4	ILD Lung	51 yr	Male	African American
H5	IPF Lung	75 yr	Female	Caucasian

Supplementary Table 2. Multiplex assessment of additional secreted proteins in the senescent and non-senescent AEC conditioned medium.

Protein	Concentration (pg/mL)		Concentration SEM (±)		Concentration/CellTiter-Glo (Ratio)		Senescence increase (Fold)	*P*-value (Paired *t*-test)
	Non-senescent AECs	Senescent AECs	Non-senescent AECs	Senescent AECs	Non-senescent AECs	Senescent AECs		
sCD40L	31.8	26.4	11.8	9.8	1.82E-05	5.82E-05	3.2	0.105
EGF	6082.8	8968.3	474.1	799.6	4.11E-03	2.32E-02	5.6	0.015
Eotaxin	11.1	8.2	3.5	1.5	6.18E-06	1.93E-05	3.1	0.029
FGF-2	781.5	317.4	341.8	295.1	4.79E-04	1.50E-03	3.1	0.482
G-CSF	1591.4	1592.2	526.1	763.7	1.24E-03	2.71E-03	2.2	0.199
GM-CSF	1151.7	529.4	206.0	288.3	7.21E-04	1.04E-03	1.4	0.090
GROα	752.3	443.8	90.2	211.9	4.64E-04	8.02E-04	1.7	0.135
IL-1α	1312.2	2435.7	186.5	729.6	9.56E-04	5.93E-03	6.2	0.014
IL1RA	64.6	25.8	28.7	4.9	3.00E-05	6.61E-05	2.2	0.387
IL-9	13.2	12.3	0.5	4.6	4.61E-06	1.62E-05	3.5	0.435
IL-12p40	9.4	11.0	1.1	0.5	3.27E-06	1.35E-05	4.1	0.088
IL-22	20.3	22.7	0.2	5.8	7.12E-06	2.93E-05	4.1	0.346
IL-27	20.2	19.7	1.7	1.2	6.53E-06	2.20E-05	3.4	0.003
IP10	6.3	5.1	2.2	1.2	3.39E-06	1.25E-05	3.7	0.036
MCP-1	1347.3	226.1	433.1	91.4	7.20E-04	5.74E-04	0.8	0.307
MCP-3	12.1	9.9	3.2	0.1	4.21E-06	1.25E-05	3.0	0.035
M-CSF	325.3	149.0	98.9	22.5	1.35E-04	1.78E-04	1.3	0.098
PDGF-AB/BB	89.9	42.6	31.9	1.2	4.66E-05	1.14E-04	2.5	0.267
TGFα	384.8	166.8	30.9	53.8	2.57E-04	3.41E-04	1.3	0.263
TNFα	282.1	54.2	72.1	24.2	1.69E-04	1.22E-04	0.7	0.019
TNFβ	8.4	5.6	1.0	2.1	5.20E-06	1.11E-05	2.1	0.065
VEGFA	117.7	14.1	47.4	5.3	5.10E-05	1.63E-05	0.3	0.093

The data presented reflects the average secreted concentration and SEM of 3 AEC donors, each with 3 technical well-level replicates per condition. Well-level replicates were first normalized to their matched CellTiter-Glo value prior to averaging to yield one value per donor, then the 3 AEC donors were averaged. Fold increase with senescence was conducted on the final ratios and a paired *t*-test was performed in GraphPad Prism. Proteins must have been within the linear range of detection in at least 2 of 3 donors to be included for the ratio and fold increase analysis. Not detected (below limit of quantitation): FLT-3L, Fractalkine, IFN-α2, IFNγ, IL-2, IL-3, IL-4, IL-5, IL-7, IL-10, IL-12p70, IL-13, IL-15, IL-17A, IL-17E, IL-17F, IL-18, MDC, MIG, MIP-1α, MIP-1β, PDGF-AA. Not included in panel: RANTES, IL-1β, IL-6, IL-8.

Supplementary Table 3. TaqMan probe identifiers for human gene expression profiling.

Species	Gene	TaqMan Probe ID
Human	β2M	Hs00187842_m1
Human	GAPDH	Hs02788624_g1
Human	SFTPC	Hs00161628_m1
Human	CAV1	Hs00971716_m1
Human	MUC1	Hs00159357_m1
Human	KRT18	Hs02827483_g1
Human	EPCAM	Hs00901885_m1
Human	CDH1	Hs01023894_m1
Human	CDH11	Hs00901479_m1
Human	SERPINE1	Hs00167155_m1
Human	KRT8	Hs01595539_g1
Human	KRT17	Hs00356958_m1
Human	KRT5	Hs00361185_m1
Human	LAMB1	Hs01055960_m1
Human	OCLN	Hs05465837_g1
Human	CLDN1	Hs00221623_m1
Human	VIM	Hs00958111_m1
Human	FN1	Hs01549976_m1
Human	CDH2	Hs00983056_m1
Human	CDKN1A	Hs00355782_m1
Human	CDKN2A	Hs00923894_m1
Human	COL3A1	Hs00943809_m1
Human	COL4A1	Hs00266237_m1
Human	TIMP1	Hs01092512_g1
Human	CCL2	Hs00234140_m1
Human	ACTA1	Hs00559403_m1
Human	CTHRC1	Hs00298917_m1
Human	FN1	Hs01549976_m1
Human	COL1A1	Hs00164004_m1
Human	PPIA	Hs04194521_s1
Human	CD44	Hs01075864_m1
Human	ACTA2	Hs00426835_g1
